# Shining Light on Anion-Mixed Nanocatalysts for Efficient Water Electrolysis: Fundamentals, Progress, and Perspectives

**DOI:** 10.1007/s40820-021-00785-2

**Published:** 2022-01-03

**Authors:** Yaoda Liu, Paranthaman Vijayakumar, Qianyi Liu, Thangavel Sakthivel, Fuyi Chen, Zhengfei Dai

**Affiliations:** 1 State Key Laboratory for Mechanical Behavior of Materials, Xi’an Jiaotong University, Xi’an, 710049 People’s Republic of China; 2 State Key Laboratory of Solidification Processing, Northwestern Polytechnical University, Xi’an, 710072 People’s Republic of China

**Keywords:** Multianions, Electrocatalysts, Water electrolysis, Hydrogen energy, Oxygen evolution

## Abstract

**Highlights:**

- This review introduces recent advances of various anion-mixed transition metal compounds (e.g., nitrides, halides, phosphides, chalcogenides, (oxy)hydroxides, and borides) for efficient water electrolysis applications in detail.
- The challenges and future perspectives are proposed and analyzed for the anion-mixed water dissociation catalysts, including polyanion-mixed and metal-free catalyst, progressive synthesis strategies, advanced in situ characterizations, and atomic level structure–activity relationship.

**Abstract:**

Hydrogen with high energy density and zero carbon emission is widely acknowledged as the most promising candidate toward world's carbon neutrality and future sustainable eco-society. Water-splitting is a constructive technology for unpolluted and high-purity H_2_ production, and a series of non-precious electrocatalysts have been developed over the past decade. To further improve the catalytic activities, metal doping is always adopted to modulate the 3*d*-electronic configuration and electron-donating/accepting (e-DA) properties, while for anion doping, the electronegativity variations among different non-metal elements would also bring some potential in the modulations of e-DA and metal valence for tuning the performances. In this review, we summarize the recent developments of the many different anion-mixed transition metal compounds (e.g.*,* nitrides, halides, phosphides, chalcogenides, oxyhydroxides, and borides/borates) for efficient water electrolysis applications. First, we have introduced the general information of water-splitting and the description of anion-mixed electrocatalysts and highlighted their complementary functions of mixed anions. Furthermore, some latest advances of anion-mixed compounds are also categorized for hydrogen and oxygen evolution electrocatalysis. The rationales behind their enhanced electrochemical performances are discussed. Last but not least, the challenges and future perspectives are briefly proposed for the anion-mixed water dissociation catalysts.

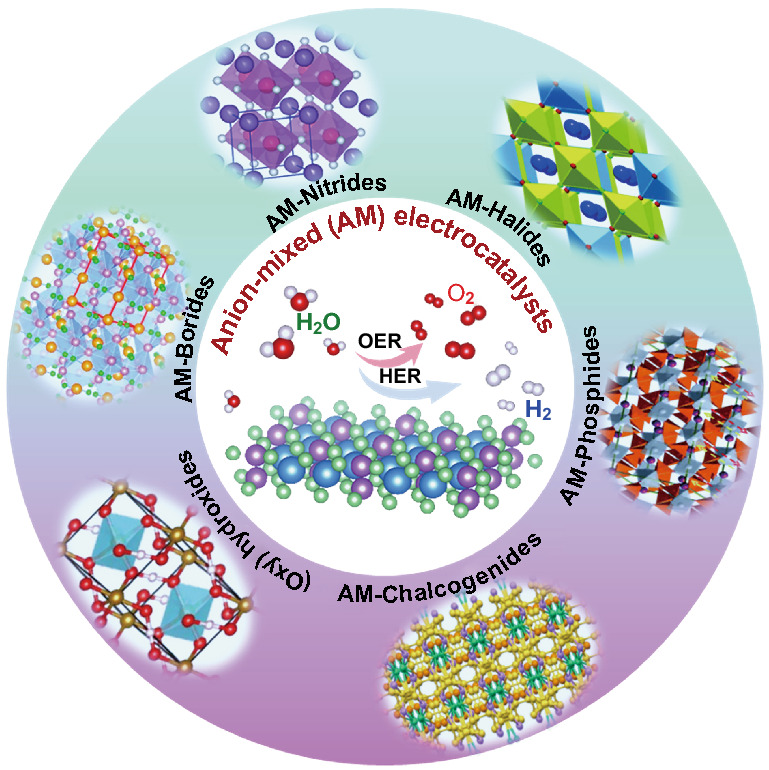

## Introduction

In the past few decades, the rapidly expanding utilization of fossil fuels has caused serious environmental issues [1]. In this regard, searching and developing alternative green and renewable energy with low-cost and long-term durability is the most enchanting research area. Among the numerous alternative energy approaches, high-purity hydrogen (H_2_, produced by photoelectrochemical and electrochemical water-splitting) serves as an economic, non-toxic, easily available, and abundant energy source due to zero emission of carbonaceous species [2–5]. As one of the most prospective techniques, photoelectrochemical water-splitting requires devices with a larger electrode area to deliver an equal volume of gas resulting in limitations while choosing feasible catalysts. By contrast, electrochemical water-splitting exhibits attractive, promising, and reliable future energy technology due to its high efficiency and convenience [6–11].

**Fig. 1:**
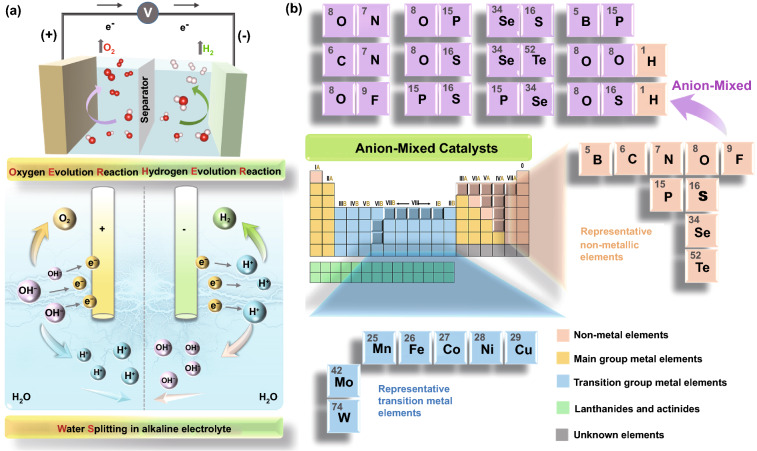
**a** Illustration of the device and mechanism of electrocatalytic water-splitting. **b** Overview of the representative elements constituting the water-splitting catalyst and the current anion-mixed pairs

In general, the electrochemical water-splitting reaction (Eq. 1) consists of two critical parts [12], hydrogen evolution reaction (HER, Eq. 2) on the cathode and oxygen evolution reaction (OER, Eq. 3) on the anode (Fig. 1a). The corresponding water-splitting reactions (i.e., alkaline solution) are as follows [13]:

1 $${\text{Overall reaction}}{:}{\text{ 2H}}_{{2}} {\text{O}}_{{({\text{l}})}} \to {\text{ 2H}}_{{{2}({\text{g}})}} + {\text{O}}_{{{2}({\text{g}})}}$$

2 $${\text{Cathode}}{:}{\text{ 2H}}_{{2}} {\text{O}}_{{({\text{l}})}} + {\text{2e}}^{-} \to {\text{ H}}_{{{2}({\text{g}})}} + {\text{2OH}}^{-}_{{({\text{aq}})}}$$

3 $${\text{Anode}}{:}{\text{ 4OH}}^{-}_{{({\text{aq}})}} \to {\text{2H}}_{{2}} {\text{O}}_{{({\text{l}})}} + {\text{O}}_{{{2}({\text{g}})}} + {\text{4e}}^{-}$$

Regardless of the electrolyte media, the standard thermodynamic potential of 1.23 V (vs. reversible hydrogen electrode, RHE) is required to split water into H_2_ and O_2_. However, both the cathode and anode reactions involve multiple electron transfer steps, and additional energy is needed to overcome kinetic obstacles and accelerate electron transfer, resulting in the so-called overpotential (*η*). Consequently, cost-effective water-splitting requires highly active and robust electrocatalysts that can significantly stimulate the kinetics of the essential two half-cell reactions. In brief, an excellent catalyst must meet two primary necessities: (i) the catalyst for per half-reaction must be highly efficient, with which small overpotential can generate high current density; (ii) the catalyst must display high durability. The fabrication of the most efficient and durable HER/OER electrocatalysts is of commercial importance to lessen the cost. The following key parameters are to be fulfilled during the fabrication of an efficient and durable electrocatalyst [14–23]:

(i) Large number of surface-active sites.
(ii) High electronic conductivity.
(iii) High intrinsic activity.
(iv) Suitable specific adsorption/desorption energy of the intermediate species.

So far, noble metal iridium(IV) oxide (IrO_2_), ruthenium(IV) oxide (RuO_2_), and platinum-based electrocatalysts are commonly used in alkaline/acidic conditions due to their high electrocatalytic activity, unique electronic properties, and long-term durability [7, 12]. Nevertheless, the large-scale commercialization of noble metals for water electrolysis is severely hindered by their scarcities and instability in electrolytes at high potentials. Given this, transition metal (TM)-based oxides, carbides, nitrides, sulfides, phosphides, chalcogenides, hydroxides, halides, alkoxides, and borides/borates have been explored [24–38]. Unfortunately, most of these materials exhibit a low catalytic performance, impecunious electronic conductivity, passivation, and dissolution under strongly acidic conditions, hindering their practical application [21, 22, 39]. Thus, advanced modification strategies are urgently needed to stimulate the activity of the catalyst, including fabricating heterostructure, modulation of the morphology, defect-rich construction, manufacturing core–shell structures, anion doping, etc. [19, 21, 40–43]. Among them, anion strategies (substitution, modulation, etc.) have shown their great potential in HER and OER. In particular, anion-mixed strategy is identified to be an exceptionally promising route in catalysts development.

Here, we summarize the latest developments in non-precious metal-based electrocatalysts for HER and OER with the anion-mixed strategy. The advantages of anion-mixed strategies to achieve superior catalytic performance are also introduced. This review starts with introducing anion-mixed electrocatalysts and the role of mixed anions, followed by recent approaches on the HER, OER, and overall water-splitting electrocatalysts, including mixed nitrides, mixed halides, mixed phosphides, mixed chalcogenides, (oxy)hydroxides, and mixed borides/borates. Figure 1b shows the representative elements constituting the water-splitting catalyst and the current anion-mixed pairs. Finally, the summary and future perspectives of the anion-mixed electrocatalysts for efficient water-splitting are also suggested.

## Anion-Mixed Electrocatalysts

Combining or mixing multiple anions is a promising way, and this emerging strategy will promote the development of non-precious materials with chemical complexity and structural diversity that are beneficial for HER, OER, and overall water-splitting [50–52]. Notably, owning to the controllable anion components and relative ratios, the anion-mixed compounds would be endowed with adjustable electronic structure, controllable intermediate adsorption/desorption energy, and tunable reaction pathway. Therefore, they are more feasible to further improve the intrinsic catalytic activity, durability, and stability than single-anion materials [34, 53, 54]. Specifically, anion mixing usually regulates the physicochemical properties of materials through the following four aspects, so as to further activate their electrocatalytic properties.

### Electronegativity Regulation

**Fig. 2:**
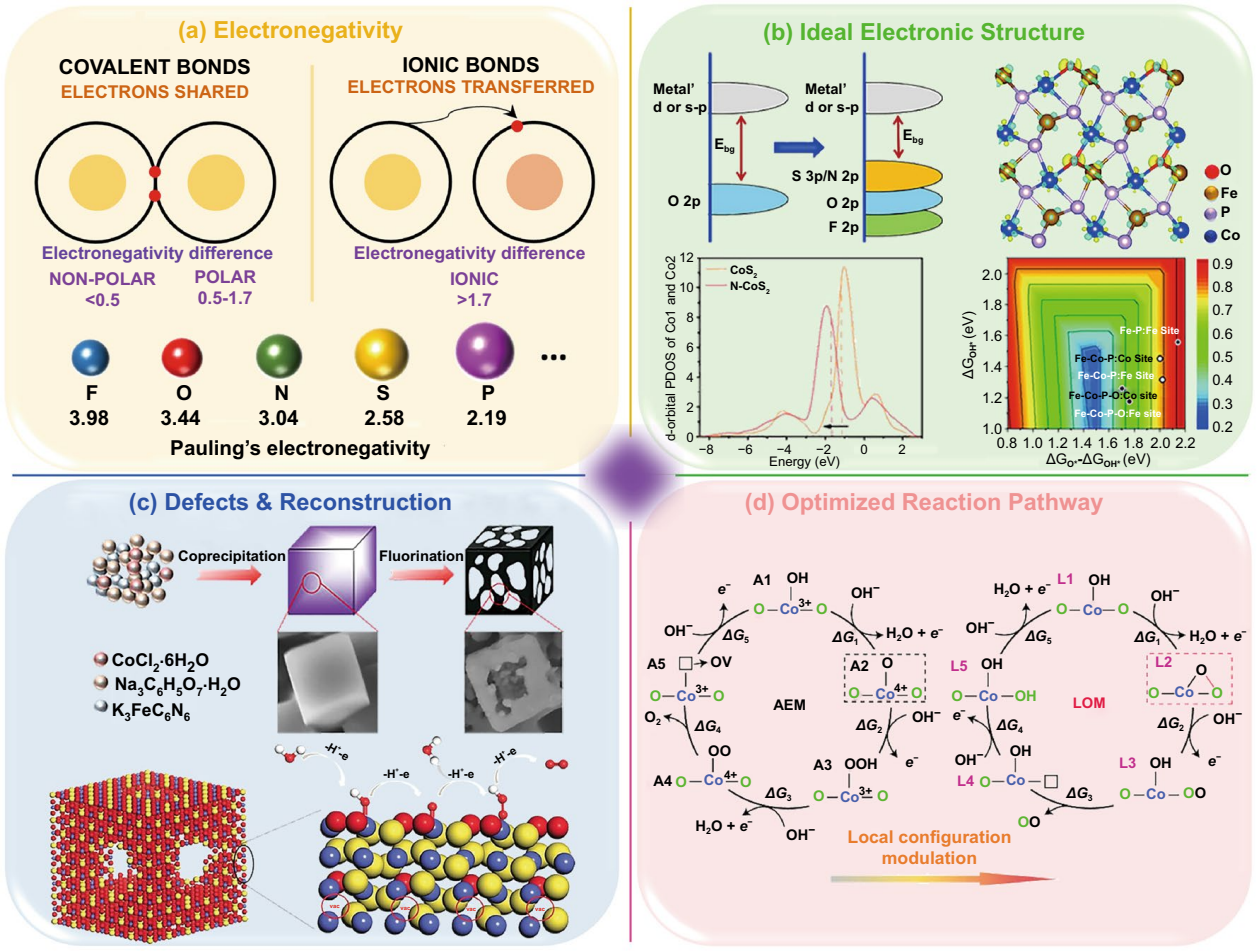
Schematic illustration of the significant features of anion-mixed regulation involved in transition metal compounds for water-splitting. **a** Electronegativity. **b** Ideal electronic structure: Schematic illustration of the strategy for band gap narrowing through anion substitution (top left); The *d*-orbital partial density of states (*d*-pdos) of Co in CoS_2_ and N–CoS_2_ (bottom left); Calculated redistribution of charge density for Fe–Co–P–O (top right, the yellow isosurface indicates electron accumulation, while the blue one represents electron depletion); Calculated volcano plot of OER overpotential *η* with ΔG_OH*_ and ΔG_O*_ – ΔG_OH*_ as the descriptors (bottom right). Reprinted with permission from Ref. [44]. Copyright Year 2019, Wiley–VCH Verlag GmbH & Co. KGaA, Weinheim. Reprinted with permission from Ref. [45]. Copyright Year 2019, Wiley–VCH Verlag GmbH & Co. KGaA, Weinheim. Reprinted with permission from Ref. [46]. Copyright Year 2019, Royal Society of Chemistry. **c** Defects and reconstruction: Schematic illustration of the synthesis process of Fe–Co–F samples (top); Schematic illustration of the oxygen-containing amorphous cobalt sulfide porous nanocubes with Co–S dangling bands (bottom). Reprinted with permission from Ref. [47]. Copyright Year 2019, Elsevier. Reprinted with permission from Ref. [48]. Copyright Year 2017, Wiley–VCH Verlag GmbH & Co. KGaA, Weinheim. **d** Optimized reaction pathway: Proposed OER mechanisms of ZnCo–OOH, including AEM and LOM. A2 and L2 are the isomeric intermediates that differentiate between the OER pathways AEM and LOM. Reprinted with permission from Ref. [49]. Copyright Year 2019, Springer Nature

Electronegativity is one of the most significant fingerprint features for anions, which contains intrinsic physicochemical information (Fig. 2a) [55]. Mixing different anions can efficiently regulate the properties and strength of bonding and bring new flexibility to material design and functionalization, such as improving conductivity, balancing the electronic structures of active centers, enhancing structural stability, etc. [56, 57]. For example, due to the strong ionic nature of the metal hydroxy fluoride (M-(OH)F) bond, F ion substitution is considered to be a powerful method to improve the catalytic activity, surface polarity, and kinetics of metal hydroxides [58]. In addition, the NiFeS system obtained by sulfurizing the NiFe layered double hydroxide precursor showed excellent OER performance because the polarized S and non-polarized O anions uniformly adjust the electronic structure of the active center [59].

Besides, the conductivity indirectly affects the activity of the catalyst, while the conductivity is directly related to the bonding polarity. For example, without using organic surfactants, the introduction of F can enhance charge mobility and improve weak conductivity [60]. Furthermore, anion diffusion exists in some solids. The advantage of anion-mixed materials is to allow one anion with stronger ionic and less charge to diffuse (improve conductivity) while maintaining the stability of the other anion with stronger covalent and charge (maintaining structural stability) [56].

Interestingly, the regulation of electronegativity also plays a positive role in ameliorating stability. For example, compared with NiCo_2_S_4_, the mixing of OH ligand with stronger electronegativity and S ligand in NiCo_2_(SOH)_*x*_ makes OH an electron acceptor, which pushed electrons from the antibonding orbital of M–S bonds to M–O bonds, so that the M–S bond in NiCo_2_(SOH)_*x*_ becomes shorter and more stable than that in NiCo_2_S_4_ [61]. This effect makes NiCo_2_(SOH)_*x*_ catalyst owns good stability in long-term work.

### Ideal Electronic Structure

According to Sabatier's principle, the H* intermediate should have appropriate adsorption strength to facilitate the HER process [62]. Similarly, considering the adsorbate evolution mechanism (AEM), OER process is accompanied by the evolution of intermediates from OH* to O*, OOH*, and O_2_ [63]. Given these, the key to improving catalyst performance is to optimize the adsorption/desorption behavior of intermediates on the catalyst surface, which is essentially achieved by adjusting the electronic structure of active sites (Fig. 2b).

The differences in radius, electronegativity, ionization potential, and electron affinity between various anions make it more possible to adjust the electronic structure [44, 64–66]. As a typical example, O incorporation in MoS_2_ could promote the hybridization between Mo *d*-orbitals and S *p*-orbitals, resulting in a smaller band gap, better conductivity, and lower energy barrier than the original 2H–MoS_2_ [67]. In addition, when S in CoS_2_ was replaced by P, the antibonding orbital was depleted, and the metal–ligand bond was strengthened, thereby enhancing the stability. The hydrogen adsorption free energy (△G_H*_) could be more thermally neutral by changing the S/P ratio, thus improving HER activity [68]. Furthermore, Fe–Co–P nanoboxes gradually transform into amorphous metal oxides during OER reaction in an alkaline medium [46]. The O and P bridges can reduce the *e*_g_ occupancy and the intermediates' free energy, thus leading to the promoted OER activity (Fig. 2b).

It is worth noting that the *d*-band center theory has been a hot research direction in the field of catalysis. The higher (lower) *d*-band center will have a stronger (weaker) affinity for the adsorbate [69]. For example, N-doping effectively adjusted the *d*-band center of CoS_2_, thus optimizing △*G*_H*_ and water adsorption Gibbs free energy (△G_H2O*_) and accelerating HER process in alkaline electrolytes [45].

### Defects and Reconstruction

Besides enhancing the intrinsic catalytic performance of the original active site, anion-mixed will obtain more active sites by constructing defects, forming lattice distortion, and promoting surface reconstruction, which is another crucial way to improve catalytic performance (Fig. 2c). For example, fluorine and oxygen can more easily replace each other because they have similar ionic radii. Based on this, the constructed NiFe oxyfluoride (NiFeOF) porous film exhibited a huge interconnected porous structure. The fluorine etching effect led to the exposure of active sites and increased the active surface area, significantly improving the electrochemical performance and further reducing the limit potential [29]. Many oxyfluorides exhibit amorphous phases, and this disordered atomic mixing mode leads to a non-equilibrium bonding environment, resulting in more active sites [70]. Besides, anion-mixed strategy based on fluorination has also been used to construct other anion defects, such as highly exposing the S-edge active site of MoS_2_ [71].

It is worth pointing out that anion substitution with different radii will cause lattice distortion. In molybdenum sulfide selenide (MoS_2*x*_Se_2(1–*x*)_) alloy, S was replaced by Se, and its incorporation increased the layer spacing (atom size of Se is larger than that of S), changed the edge electronic structure, and achieved high conductivity and narrow band gap, thus improving HER performance [72].

Furthermore, anion-mixed can promote surface reconstruction. As a typical example, iron–cobalt–fluoride (Fe–Co–F) nanocubes with abundant defects/voids were formed through fluoride etching [47]. The increased electrochemical specific surface area caused by surface roughness and the metal oxides easily formed on the catalyst surface make this new catalyst very active and stable for water oxidation. These advantages benefit from the surface reconstruction caused by the coexistence of metal–O and metal–F bonds. Similarly, amorphous CoO_0.6_S_4.6_ porous nanocube was prepared by mixing O and S anions [48]. Due to the embedding of O atoms around the Co center, the local disordered structure was formed. There were 4.6 S atoms and 0.6 O atoms around the Co atom, forming abundant Co–S dangling bonds. Oxygen doping on CoS_*x*_ support and Co–S hanging bond can greatly enhance the adsorption of O*.

### Optimized Reaction Pathway

It is well known that both HER and OER are processes involving multiple electron steps. Therefore, advanced catalysts should not only be beneficial to the adsorption of a single intermediate but also coordinate and optimize the evolution process of each intermediate to minimize the energy of the rate-determining step (Fig. 2d). Anion-mixed strategy is a vital means to achieve this goal. For example, in alkaline conditions, under alkaline conditions, Δ*G*_H*_ is not the only descriptor for HER process, and the dissociation energy barrier of water plays a decisive role. Studies have shown that P doping reduced the electron density around Ni atoms and increased the charge of Se, thus reducing the energy barrier of hydrolysis [73].

As another typical example, the synergistic effect of B and P was studied in amorphous Co_2.90_B_0.73_P_0.27_ ternary alloy [74]. Element P could accelerate the dissociation of H_2_O and promote the Volmer process. For Co–P system, H was strongly bound to Co^δ+^, which was not conducive to H_2_ overflow. The addition of B in Co–B–P system made the binding energy of Co shift negatively, optimizing the H–Co interaction and promoting the Heyrovsky process. In addition, the “dual ligand coordinated modulation” strategy has realized the optimization of the OER reaction pathway. For example, the synergistic effect of OH and S ligands on the surface of NiCo_2_(SOH)_*x*_ subtly adjusted the electronic structure and chemical environment of active metal centers, thus optimizing the binding energy of OER intermediates (OH*, O* and OOH*) synchronously, which is helpful to meet the energy requirements of OER evolution process [61].

Interestingly, (oxy)hydroxides are special anion-mixed OER catalysts. Before the start of OER, the transition metal center usually undergoes an electrochemical pre-oxidation process and deprotonation. Due to the structural flexibility and highly oxidized transition metal center, many (oxy)hydroxides are prone to form O–O bonds directly, which will trigger the lattice oxygen oxidation mechanism (LOM) to obtain higher OER activity [75]. The introduction of Zn^2+^ in CoOOH has also been proved to effectively control the mechanism switching of catalysts in OER process [49].

Based on these advantages, in the following sections, the anion-mixed strategies applied in developing such electrocatalysts and their outstanding performances in water-splitting are described in detail.

## Electrocatalytic Performance of Anion-Mixed Electrocatalysts

### Mixed Nitrides

#### Oxynitride

The incorporation of nitrogen (N) into oxides opens the facile route of tuning physical and chemical properties [76–78]. As N with weaker electronegative and is more polarizable than O, the M–N bond is more covalent than the M–O bond in nature [79]. Besides, N has a higher ionic radius than O, the substitution of N into the oxide makes an increase in bonding distance [80]. Thus, regulating the N ratio in TM-based compounds can modify their structure, improve the charge transfer process, and lead to enhanced electrocatalytic activities [81, 82]. Notably, combining transition metals (TMs) with oxynitrides is also an efficient and viable method to enhance its electrical conductivity and corrosion resistance [83].

Given these advantages, Miura et al. investigated the regulation of N ratio and electronic structure with a correlated electrocatalytic performance of manganese oxynitride [87]. Catalytic performance of N-rich manganese (Mn) oxynitrides can be enhanced by nearly single-electron occupancy of the antibonding e_g_ states and highly covalent Mn–N bonding. Similarly, Chang et al. designed a novel vanadium–nickel oxynitride (VNiON) layer covered on the corresponding oxide nanosheets, which have been proven to exhibit improved electrochemical performances [55]. The substitution of the –2 charged oxyanion by the –3 charged nitride anion results in transition-metal oxynitride (TMON) layers owning additional metal cation valence, which helps to absorb the lone pair of nitrogen electrons [88]. The similar atomic radii of N and O also enable them to reduce the effect of superimposed lattice distortion as much as possible in the process of mutual substitution and maintain good structural stability [89].

**Fig. 3:**
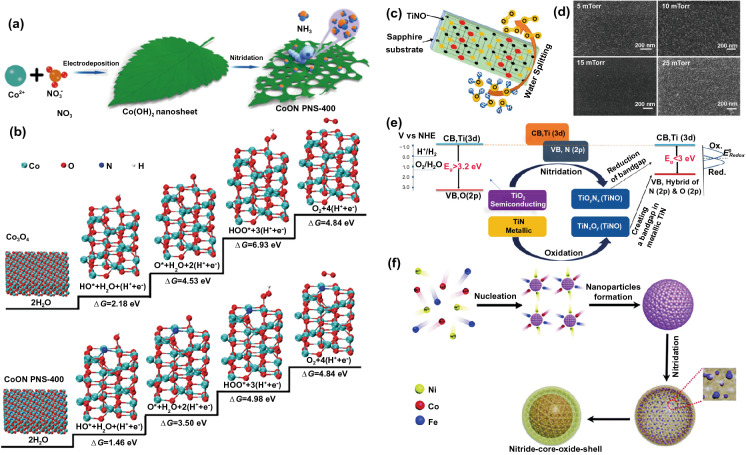
**a** The schematic illustration of the formation of CoON PNS-400 nanosheets. **b** DFT calculated free energy diagram of the OER on Co_3_O_4_ and **b** CoON PNS-400, respectively. Reprinted with permission from Ref. [84]. Copyright Year 2018, Wiley–VCH Verlag GmbH & Co. KGaA, Weinheim. **c** Schematic illustration of TiNO thin films for electrocatalytic water oxidation, **d** SEM images of TiNO films grown in oxygen pressures of 5, 10, 15, and 25 mTorr, **e** Band diagram showing a downward movement of VB via nitridation of TiO_2_ and opening of a bandgap between overlapping valence and conduction band in a metallic TiN via oxidation of TiN. Reprinted with permission from Ref. [85]. Copyright Year 2020, American Chemical Society. **f** Illustration of the formation of nitride-core, oxide-shell FeCoNi oxynitride nanoparticles. Reprinted with permission from Ref. [86]. Copyright Year 2019, Royal Society of Chemistry

Recently, Lei's group developed porous cobalt oxynitride nanosheets (CoON PNS) for OER electrocatalysis (Fig. 3a) [84]. Specifically, this CoON PNS exhibited a low overpotential (*η*_10_) of 0.23 V at a current density (*j*) of 10 mA cm^–2^ for the OER, which is much better than that of Co_3_O_4_ (0.34 V) nanosheets (NSs). Density functional theory (DFT) calculation revealed that the free energy for HO* (∆*G*_HO*_) and O* (∆*G*_O*_) formation on CoON PNS-400 is 1.46 and 3.50 eV, respectively, noticeably smaller than that of Co_3_O_4_ nanosheets with ∆*G*_HO*_ = 2.18 eV and ∆*G*_O*_ = 4.53 eV (Fig. 3b). More crucially, the free energy significantly reduces from 6.93 to 4.98 eV, supremely can be considered as the rate-controlling step of water oxidation (HOO* formation). The above theoretical results prove the inclusion of foreign N atoms into Co_3_O_4_ effective regulation of the free energies toward an optimal value. Especially, TiN_*x*_O_*y*_ (TiNO) films were in situ synthesized in the oxygen partial pressure range of 5–25 mTorr by pulsed laser deposition (Fig. 3c, d) [85]. The electrochemical overpotential of TiNO film for water oxidation is as low as 290 mV (10 mA cm^–2^). The electrocatalytic performance improvement is due to the number increase of the active sites, which was realized by O replacing the anion N site and the rearrangement of the valence band relative to the redox potential of the electrolyte medium (Fig. 3e).

Moreover, bimetallic/trimetallic catalysts have been given significant attention because of the multiple active species, enhanced charge transfer between different ions, and capability of the electronic structure tune at the electrode surface/interface. Taking advantage of bimetallic and anion-mixed strategy, Song's group fabricated a 3D bifunctional nanohybrid electrode based on cobalt nitride–vanadium oxynitride on carbon cloth (CVN/CC) toward overall water-splitting [90]. CVN/CC achieved *η*_10_ = 0.263 V for OER and *η*_10_ = 0.118 V for HER in Ar-saturated 1.0 M KOH solution. In addition, it exhibited overall water-splitting with a cell voltage of 1.64 V at a *j* of 10 mA cm^–2^ in alkaline media. A reported case by Dai and coworkers showed that the electrocatalytic performance of the iron–cobalt–nickel (FeCoNi) oxynitride sample (*η*_10_ = 0.291 V) outperformed that of state-of-the-art catalysts (Fig. 3f) [86]. A similar comparable result achieved by Xiong et al. showed that the combination of the excellent metal like OER activity of nitrides and the inherent oxidation resistance of oxides could obtain FeNi oxynitrides with excellent performance, which has a low overpotential of 295 mV (10 mA cm^–2^, 1 M KOH) and considerable durability [91]. The synergistic interaction between Fe and Ni components creates a favorable local coordination environment for OER. These results suggest that the unique properties of oxynitrides bring excellent performance in water-splitting reactions.

#### Carbonitride

Among the high-performing several mixed nitrides, carbonitrides are considered effective and promising electrocatalysts due to their desirable electronic structures, and their density of states (DOS) around the Fermi levels is the same as that of the precious metals. The *p*–*d* state equilibrium between TM and carbon or nitrogen atoms can be adjusted by additional elements, which can significantly affect its macro-mechanical properties, microstructure, and electronic stability.

**Fig. 4:**
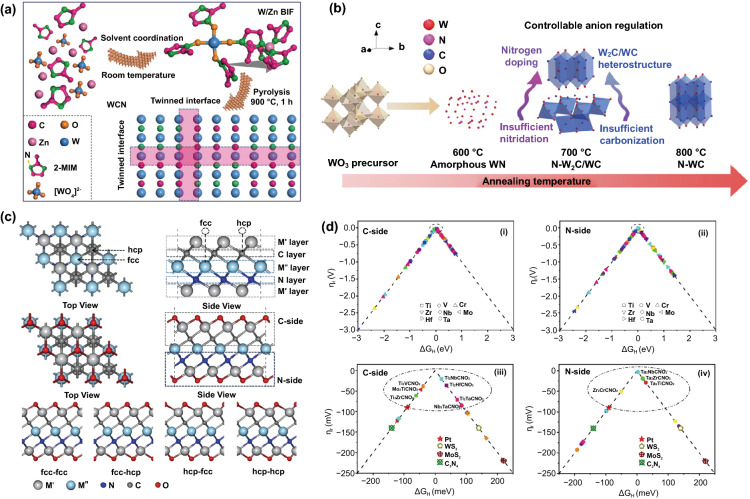
**a** Schematic diagram of the construction method for twinned WCN. Reprinted with permission from Ref. [92]. Copyright Year 2019, Wiley–VCH Verlag GmbH & Co. KGaA, Weinheim. **b** Schematic representation of the controllable anion regulation. Reprinted with permission from Ref. [93]. Copyright Year 2020, American Chemical Society. **c** The bare structure of ordered double transition metal carbonitrides, **d** The volcano plotted for all 64 M′_2_ M″CNO_2_-MXenes. (i) C-side of M′_2_ M″CNO_2_-MXenes. (ii) N-side of M′_2_ M″CNO_2_-MXenes. (iii) The corresponding enlarged view of the area near the volcano apex of C-side. (iv) The corresponding enlarged view of the area near the volcano apex of N-side. The different shapes represent different M′, and different colors represent different M″ (red, orange, yellow, green, cyan, blue, purple, and pink represent Ti, V, Cr, Zr, Nb, Mo, Hf, and Ta, respectively). Reprinted with permission from Ref. [94]. Copyright Year 2021, Springer Nature

For example, Kou et al. explored the WCN with a twinned structure that exhibited outstanding electrocatalytic activity (*η*_10_ = 140 mV) for HER as compared to the W_2_(C, N) (Fig. 4a) [92]. Notably, the intergrown of W_2_C and WN in the WCN nanocrystals yields abundant N–W–C interfaces, driving a notable improvement in high electrocatalytic HER. Besides, 3D flower-like N-doped tungsten carbide (W2C/WC) with rationally regulated anions was prepared by annealing at a high temperature of 600–800 °C in Ar atmosphere (Fig. 4b) [93]. Interestingly, adjusting annealing temperature would change orthorhombic/hexagonal (W_2_C/WC) structure and crystallographic phases. The W–C and W–N ratios can also be subsequently modulated. This N-doped W_2_C/WC-700 °C heterostructure array showed a low overpotential of 0.063 V at a current density of 10 mA cm^–2^ and a small Tafel slope of 73 mV dec^–1^.

The regulation of N doping also manages the improvement in electronic conductivity and electrochemical performance of Mo-based compounds. For example, Nakanishi et al. have developed MoCN nanoparticles by using in situ CO_2_ emission strategy and the highly abundant amino group-based polydiaminopyridine (PDAP), polyaniline (PANI) precursors, which performed a high H_2_ production rate (at – 0.14 V vs. RHE, 10 mA cm^–2^) [95]. Besides, Wei et al. synthesized 3D nitrogen-doped flower-like carbon nanospheres (NFCNS) loaded with nitrogen-doped molybdenum carbide (N–Mo_2_C) nanoparticles through cationic surfactants [96]. Nitrogen atoms are doped into the lattice and carbon skeleton of Mo_2_C at the same time, resulting in low desorption energy of the Mo–H bond and easy hydrogen evolution. In addition to experimental means, Li's group studied the HER activities of 64 O-terminated ordered double transition metal carbonitrides (M′_2_ M″CNO_2_, M′ = Ti, V, Cr, Zr, Nb, Mo, Hf, Ta; M″ = Ti, V, Cr, Zr, Nb, Mo, Hf, Ta) utilizing DFT calculation (Fig. 4c) [94]. The results showed that Ti_2_NbCNO_2_ had a small hydrogen adsorption Gibbs free energy (Δ*G*_H*_, 0.02 eV), abundant C-side catalytic centers, and great stability; therefore, it is expected to be a Pt-like HER catalyst (Fig. 4d).

It is worth noting that boron carbonitride (BCN) has recently been proved to be an effective electrocatalytic catalyst with excellent stability [97, 98]. Taking representative research as an example, Lewis acid–base theory and DFT revealed that the enrichment of B/N heteroatoms in boron carbon nitride (BCN) system could lead to stronger OH*/H_2_O adsorption strength, respectively [98]. The self-supporting BCN material electrode pair (B-BCN/N-BCN) with the best B and N content was able to complete overall water-splitting at a low voltage of 1.52 V (10 mA cm^–2^). This novel work provides a valuable reference for developing advanced metal-free materials and multicomponent carbonitride materials for electrocatalytic water-splitting.

### Mixed Halides

#### Oxyfluoride

TM oxyfluorides (OF) possess unique characteristics, including optical properties, catalytic activity, and electrical conductivity [58, 99, 100]. It is interesting to note that the intrinsically weak conductivity of metal–fluorides can be improved by introducing metal–oxygen (M–O) bonds, because the high ionicity of metal–fluorine (M–F) bonds can be well adjusted by high covalent M–O bonds. Thus, M–OF contains both extraordinary conductivity and electrochemical characteristics due to the coexistence of M–O and M–F bonds [101]. It is worth noting that the F atoms possess strong electronegativity among all of the elements in the periodic table; thus, they can easily capture electrons from neighboring metal atoms, resulting in numerous coordinatively unsaturated sites [101]. Meanwhile, during exploring highly active, low-cost, and long-term durable catalysts for water-splitting, oxyfluorides have attracted much attention due to their non-centrosymmetric macroscopic polarity, magnetic properties, and redox properties [102–105].

**Fig. 5:**
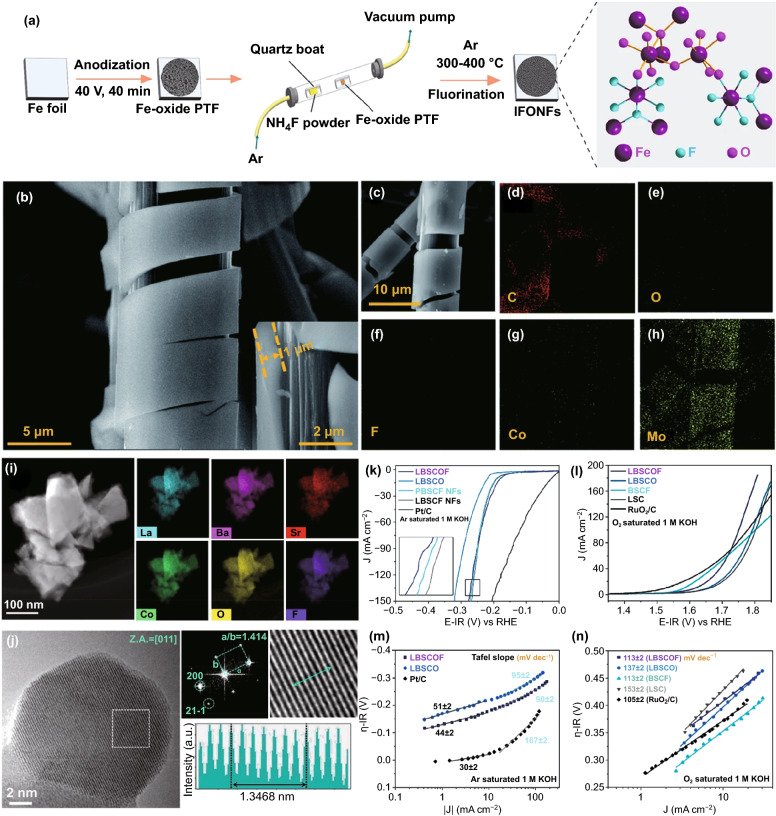
**a** Schematic of the fabrication process for IFONFs, starting with a Fe foil. Reprinted with permission from Ref. [31]. Copyright Year 2018, Springer Nature. **b** SEM images for CoMoOF/GF. Inset: cross-sectional SEM image at a higher magnification, **c–h** SEM image for CoMoOF/GF and the corresponding EDS elemental mapping. Reprinted with permission from Ref. [108]. Copyright Year 2021, Royal Society of Chemistry. **i** Scanning transmission electron microscope (STEM)-energy dispersive X-ray spectroscopy (EDX) analysis of LBSCOF shows the uniform element distributions, **j** A typical high resolution transmission electron microscope (HRTEM) image of LBSCOF and the corresponding simulation results, **k** LSV curves and **l** Tafel plots and slopes for HER in Ar-saturated 1 M KOH, **m** LSV curves and **n** Tafel plots and slopes for OER in O_2_-saturated 1 M KOH. Reprinted with permission from Ref. [104]. Copyright Year 2018, Elsevier

By employing a facile low-temperature synthetic methodology, Fan et al. developed the 3D iron fluoride-oxide nanoporous film (IFONF) with both abundant defect sites as a bifunctional electrocatalyst for water-splitting (Fig. 5a) [31]. For overall water-splitting, a *j* of 10 mA cm^–2^ can be achieved at a voltage of 1.58 V, which is lower than the benchmark of Ir/C–Pt/C couple (1.62 V). Besides, due to the highest electronegativity of the F^–^ anion (∼3.98) and the comparable ionic radii of F^–^ (1.31 Å) to O^2–^ (1.38 Å) ions, the F^–^ anion at the oxygen sites showed the anion-doping extremum and acts as a suitable *n*-type dopant for cobalt–oxide (Co–O)-based electrocatalysts. Taking this as an advantage, Huang et al. indicated that 2D cobalt–oxyfluoride with the incorporation of cobalt–fluoride (Co–F) and Co–O bonds for effectively modulated electronic structure, exhibiting an efficient electrochemical activity (*η*_10_ = 230 mV) with high durability (45 h) toward OER [106].

Multielement transition metal oxyfluorides are also the current research hotspot object [101]. For instance, Svengren et al. prepared Co_3_Sb_4_O_6_F_6_ with an electrocatalytic water-splitting activity of *η*_10_ = 0.43 V for OER [107]. Yang et al. developed a NiFe oxyfluoride (NiFeOF) holey film (HF) as an overall water-splitting electrocatalyst [29]. The as-prepared NiFeOF HF showed excellent overall water-splitting performance (*η*_10_ = 0.6 V in 1 M NaOH) due to the combination of mixed phases (crystalline NiFe alloy/amorphous NiFeOF) and porous structure. In addition, F and O can be interchanged with each other more quickly due to the similar ionic radii of O^2–^ and F^–^, providing higher electrical conductivity to enhance the electrochemical catalytic performance.

The structural advantage and the synergistic effect of the bimetal components also contribute to improving the catalytic activity. For example, Lei et al. produced amorphous CoMo bimetallic oxyfluoride (CoMoOF/GF) on graphite felt, and proper anodizing made it more abundant in defects and larger pore structure (Fig. 5b–h) [108]. CoMoOF/GF showed a quite low overpotential and Tafel slope at *j* = 10 mA cm^–2^ (79 mV, 43.3 mV dec^–1^ in the alkaline electrolyte; 94 mV, 60.2 mV dec^–1^ in the acid electrolyte). Additionally, perovskite fluoride oxide is also a typical configuration. Hua et al. reported a La_0.5_Ba_0.25_Sr_0.25_CoO_2.9–δ_F_0.1_ (LBSCOF) catalyst for alkaline water-splitting (Fig. 5i, j) [104]. For HER, the overpotential value of LBSCOF at 100 mA cm^–2^ is 256 mV (Fig. 5k). It should be noted that the Tafel slope (90 mV dec^−1^) of LBSCOF in the high current density region (> 10 mA cm^–2^) is only about half of Pt/C (167 mV dec^−1^) (Fig. 5l). For OER, LBSCOF can reach a current density of 100 mA cm^−2^ at an operating voltage of 1.748 V, and the Tafel slope is 113 mV dec^−1^ (Fig. 5m, n). F anion helps to enhance the center of the O *p* band and activate the redox ability of lattice O.

#### Hydroxy Fluoride

Recently, F anion substituting was found as an assuring to increase the catalytic activity, surface polarity, and kinetics of the metal hydroxyl oxide because of the high ionicity of metal hydroxyl fluoride (M-(OH)F) bond. Interestingly, the inclusion of F anions into the host hydroxyl oxide lattice improved the polarity of the chemical bonds, which enhanced the adsorption capacity of hydroxide (OH) anions and oxygenic intermediates [58].

**Fig. 6:**
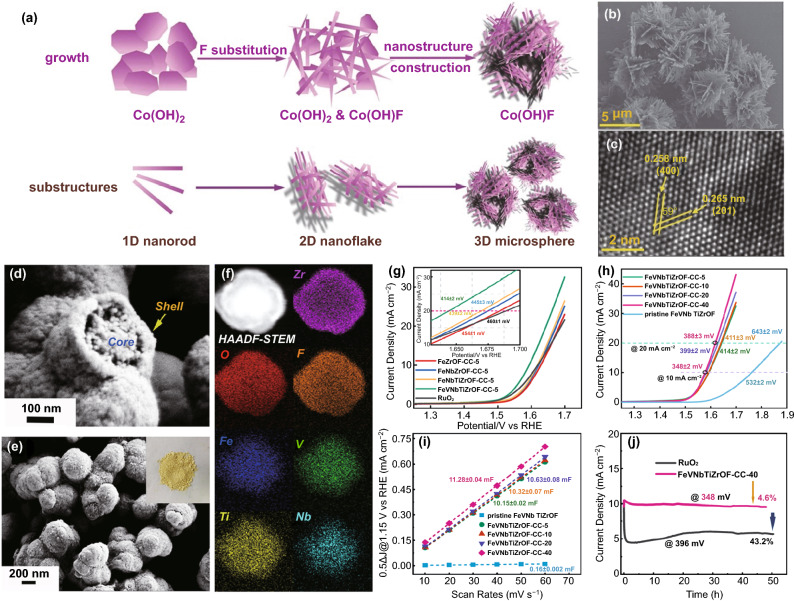
**a** Schematic of the growth and substructures of the hierarchical 3D Co(OH)F microspheres. The 3D structure is constructed by 2D networked nanoflakes, and the 2D nanoflakes are further woven by the 1D nanorods, **b** SEM and **c** HRTEM images of the hierarchical 3D Co(OH)F microspheres. Reprinted with permission from Ref. [60]. Copyright Year 2017, Wiley–VCH Verlag GmbH & Co. KGaA, Weinheim. **d–e** SEM images and **f** EDS mappings of the pristine FeVNbTiZrOF, the insert map is the optical photo of the pristine FeVNbTiZrOF, **g** LSV curves of FeZrOF-CC-5, FeNbZrOF-CC-5, FeNbTiZrOF-CC-5, FeVNbTiZrOF-CC-5 and RuO_2_ in 1 M KOH solution with a scan rate of 5 mV/s; **h** LSV curves of the pristine and activated FeVNbTiZrOF at different current densities; **i** corresponding linear plots of capacitive current densities versus scan rates; **j** Chronoamperometric response of FeVNbTiZrOF-CC-40 and RuO_2_ at the overpotential of 348 mV and 396 mV, respectively. Reprinted with permission from Ref. [113]. Copyright Year 2021, Elsevier

The F-guided synthesis approach offered the possibility to improve poor conductivity without resorting to organic surfactants. For instance, Wan et al. developed the synthesis of 3D cobalt hydroxy fluoride (Co(OH)F) microspheres using F as an oxidatively robust ligand (Fig. 6a) [60]. In particular, the substitution of the fluorine anion ensured that the development of a unique 3D structure is pure orthorhombic Co(OH)F phases, with modified electronic structure contributed better charge mobility (Fig. 6b, c). Owing to these, the Co(OH)F electrode showed superior catalytic activity (10 mA cm^−2^ at 0.313 V) and excellent OER stability (more than 10 h with a *j* of 8 mA cm^−2^). Analogously, to improve charge transferability, Hu's group reported NiFe hydroxide nanosheets incorporating fluoride (NiFe-(OH)F) on Ni foam demonstrating the enhanced HER activity and lower ohmic resistance than NiFe hydroxide (NiFe–OH) catalyst [109].

In addition to adjusting the electronic state, F ions also have unique properties to promote catalyst reconstruction in the OER process. NiFe-hydroxide NS arrays on 3D NF (NiFe–OH–F) were further developed by incorporating fluoride through the one-step hydrothermal method [110]. NiFe–OH–F was converted to a highly mesoporous and amorphous metal oxide hierarchical structure through surface self-reconstruction by fluoride leaching under OER conditions. Excitingly, the resulting surface reconstructed (SR) NiFe–OH–F achieved a *j* of 10 mA cm^–2^ at a small overpotential (0.176 V) in the alkaline medium for OER. Besides, Xu et al. demonstrated a new electrochemically driven F-induced surface-reconstruction strategy for converting ultra-thin NiFeO_*x*_F_*y*_ nanosheets into Fe-enriched Ni(Fe)O_*x*_H_*y*_ phase [111]. Moreover, through a fluorination strategy, Ma et al. obtained a hierarchical NiFe Prussian blue analogue (NiFe-PBA)-F catalyst, which showed an ultra-low OER overpotential of 190 mV (10 mA cm^–2^) with excellent stability [112]. The dynamic migration of fluoride ions promotes the interesting reconstruction of NiFe-PBAs-F, presenting more F-doped NiFeOOH active sites for intermediates' adsorption.

Typically, the high entropy effect, amorphous state, and structure effect are new ideas to enhance OER performance. Zhu et al. applied chronopotentiometry to in situ activate multicomponent non-noble metal oxyfluoride as an advanced OER catalyst [113]. The increase of current density was conducive to reconstructing the catalyst's structure into a nanoporous core–shell structure and introducing metal hydroxyl oxide on the surface, which increased effective ion/mass transport channels and active catalytic sites (Fig. 6d–f). The activated five-membered oxyfluorides showed the best catalytic activity, with an overpotential of 348 ± 2 mV at a current density of 10 mA cm^–2^ (Fig. 6g, h), a double layer capacitance (C_dl_) value of 11.28 ± 0.04 mF (Fig. 6i), and stability of up to 50 h (Fig. 6j).

### Mixed Phosphides

#### Oxyphosphide

Oxygen (O) anion incorporation in metal phosphides (M–P) can further boost the intrinsic activity of electrocatalysts. Indeed, due to the firmness of M–P–O metal bonds in oxyphosphide, it has been proved to have excellent stability [114]. In addition, the metal–P (M–P) and metal–O (M–O) bonds in oxyphosphide will help to adjust the electronic structure and optimize the adsorption/desorption free energy of intermediates. And the interaction of phosphorus and oxygen produces plentiful surface vacancy/defects and large active surface area [46, 115–117]. Given these, oxyphosphides are considered highly potential water-splitting catalysts. Besides, various appearance configuration is another feature for the oxyphosphides’ family.

**Fig. 7:**
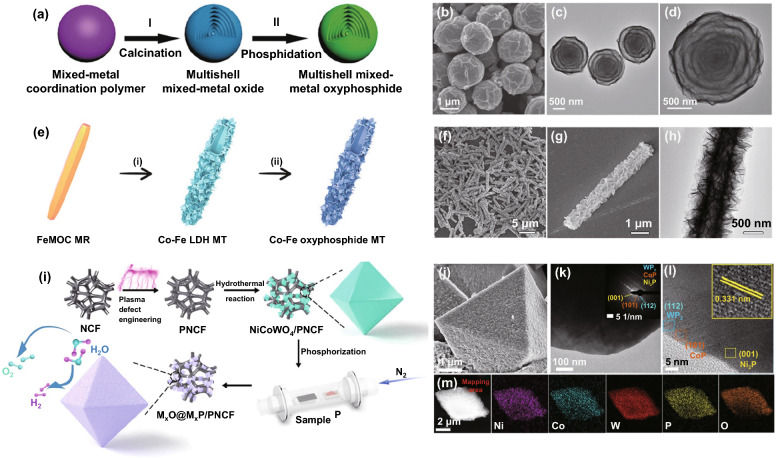
**a** Formation of multishell mixed-metal oxyphosphide particle, **b** FESEM and **c, d** TEM images of the multishell Mn–Co oxyphosphide particles. Reprinted with permission from Ref. [118]. Copyright Year 2017, Wiley–VCH Verlag GmbH & Co. KGaA, Weinheim. **e** Schematic illustration of the formation process of hierarchical Co–Fe oxyphosphide MTs, **f, g** FESEM and **h** TEM images of hierarchical Co–Fe LDH MTs. Reprinted with permission from Ref. [119]. Copyright Year 2019, Wiley–VCH Verlag GmbH & Co. KGaA, Weinheim. **i** Fabrication of a multiphase, trimetallic Ni–Co–W phosphoxide electrocatalyst: Regular octahedral multiphase M_*x*_O@M_*x*_P crystals are formed in situ on plasma-defect-engineered Ni–Co support, **j** high-resolution SEM image, **k, l** low-and high-resolution TEM images, the inset in **k** is the SAED pattern of the M_*x*_O@M_*x*_P/PNCF particle, and **m** HAADF-STEM image and EDX elemental mapping of a single M_*x*_O@M_*x*_P/PNCF. Reprinted with permission from Ref. [120]. Copyright Year 2021, American Chemical Society

Lou and his colleagues have carried out lots of typical research work on oxyphosphides with novel configurations and excellent performance. For example, they creatively designed highly complex multishell MnCo oxide particles with seven layers of shells [118]. After phosphorization treatment, the multishell were transformed into MnCo oxyphosphide particles (Fig. 7a–d), exhibiting better OER performance than previous MnCo oxide particles. The substitution of Mn^2+^ cations and O^2–^ anions into the CoP was beneficial for conductivity enhancement and the generation of new active sites. Likewise, they fabricated hierarchical CoFe oxyphosphide microtubes with hollow structures via a facile self-templated synthetic approach, including phosphorization treatment (Fig. 7e) [119]. Typically, field emission scanning electron microscopy (FESEM) and transmission electron microscope (TEM) analysis of the sample confirms the formation of the tubular structure (Fig. 7f–h). The obtained electrocatalyst exhibits overpotentials of 280 and 180 mV for OER and HER (10 mA cm^−2^, alkaline media), respectively.

Similarly, some mixed hybrid oxyphosphides with other configurations are also reported. Wang and coworkers constructed a mixed-metal–organic framework (MMOF) self-template supported CoFe hybrid oxyphosphides [8]. Significantly, due to the oxygen incorporation, Co_3_FeP_*x*_ considerably strengthened the O* adsorption, which augmented the OER activity. As a result, the mixed-metal oxyphosphides were found to have a lower overpotential 0.291 V at a *j* of 10 mA cm^−2^. Hong's group constructed a novel template-engaged technique to make bimetal oxyphosphide of different Fe ions as modulators in hollow NiFe spheres (H–NiFe) [121]. The resulting material duly showed excellent OER performance with long-term stability (20 h at a static overpotential of 0.27 V) due to the optimized chemical composition and the subtly constructed hollow structure of H–NiFe spheres. Furthermore, researchers from Du's group successfully developed the ultra-fine nickel–zinc oxyphosphide (NiZnPO) nanosheets (NSs) [122]. Interestingly, the NiZnPO NSs doped with oxyphosphide anions enhance modulated the local electronic structure, promoted active areas, as well as improved electron conductivity. The NiZnPO NSs demonstrated high activity, with an overpotential of 0.29 V required to achieve a *j* of 10 mA cm^–2^ for the OER, and showed excellent long-term stability for over 3000 cyclic voltammetric cycles. In another study, Xu et al. developed a 3D-1D CoZn oxyphosphide NSs anchored on CNTs with uniform surface coverage, displaying an excellent electrocatalytic performance toward OER at an overpotential of 0.28 V (*j* = 10 mA cm^–2^) [123]. The overpotential would be decreased significantly by the presence of 3D-1D architecture, modified electronic structure, increased surface active sites and the promotion effect of P atoms.

For practical applications, the catalyst that meets the industrial needs should have the characteristics of low overpotential (≤ 300 mV) under high current density (≥ 500 mA cm^–2^), high performance, and excellent durability. Given this, regular octahedral nickel–cobalt–tungsten–phosphorus oxide particles (MxO@MxP/PNCF (M = Ni, Co, W)) were successfully formed in situ on plasma-defect-engineered Ni–Co support and served as HER catalyst (Fig. 7i) [120]. Notably, low overpotentials of only 53 and 343 mV for current densities of 10 and 1000 mA cm^–2^ were required for H_2_ production. Notably, the strong metal oxide octahedral support is conducive to improving the electrocatalytic stability of MxO@MxP (Fig. 7j–m), which demonstrates excellent long-term stability and durability with no significant activity loss.

#### Hydroxy Phosphate

**Fig. 8:**
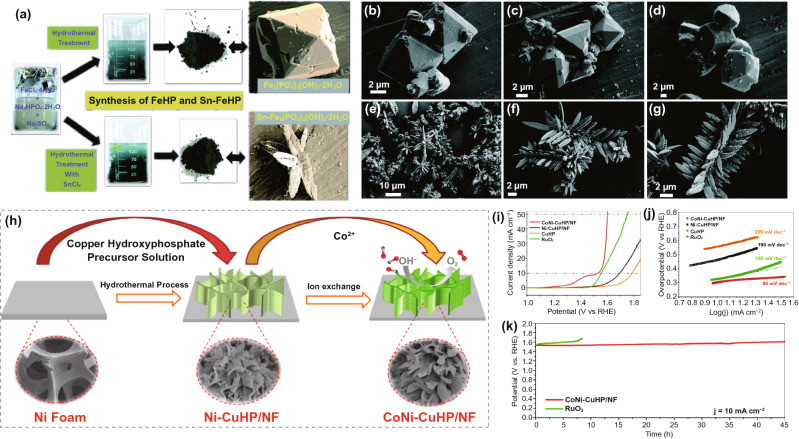
**a** The synthesis of FeHP and Sn-FeHP from iron(III) chloride, tin(II) chloride, and disodium hydrogen phosphate. **b–d** FE-SEM images of FeHP octahedral crystals, **e–g** FE-SEM images of Sn–FeHP self-assembled needle-like microcrystals. Reprinted with permission from Ref. [126]. Copyright Year 2017, Royal Society of Chemistry. **h** Schematic illustration of the synthesis of CoNi–CuHP/NF, **i** LSV curves, and **j** the related Tafel curves of CoNi–CuHP/NF and different catalysts, **k** long-term cycling tests of the CoNi–CuHP/NF catalyst and commercial RuO_2_. Reprinted with permission from Ref. [125]. Copyright Year 2017, Royal Society of Chemistry

Hydroxy phosphate (HP) is another representative oxyphosphide. As an excellent electrocatalyst, Fe_2.95_(PO_4_)_2_(OH)_2_ thin-film electrode exhibited an overpotential of 281 mV for OER (1 M KOH, 10 mA cm^–2^) and 165.7 mV for HER (1 M H_3_PO_4_, 10 mA cm^–2^), respectively [124]. As per previous reports, the TM-HP are commonly much more durable than the corresponding TM-phosphates in alkaline electrolytes [125]. For instance, Mani et al. reported orthorhombic iron hydroxy phosphate (Fe_5_(PO_4_)_4_(OH)_3_·2H_2_O (FeHP)) synthesized by hydrothermal method from metal chloride precursors and disodium hydrogen phosphate as a source of phosphate ligand precursor (Fig. 8a) [126]. To enhance the OER behavior, the authors introduced Sn into FeHP to form Sn–FeHP to weaken M^3+^–OH bonding. After Sn incorporation, the nanocrystal morphology of the FeHP was changed, showing a star-like nanostructure (Fig. 8b–g). Notably, Sn–FeHP has achieved catalytic activity with a small onset overpotential of 0.35 V at 10 mA cm^–2^ with a Tafel slope of 81 mV dec^–1^ (1 M KOH). Moreover, the SN–FeHP possessed remarkable stability in alkaline media for 800 min at 1.65 V vs. RHE. Additionally, the electrocatalytic activity of the FeHP electrode can be further developed with a combined effect of Ni or Co. As such, Fe (Ni/Co) hydroxyphosphate/NF was reported as demonstrating a bifunctional electrocatalyst [127]. Summing up, a cell voltage of just 1.65/1.67 V at a current density of 10 mA cm^–2^ was required to achieve overall water-splitting by using Fe (Ni/Co)HP/NF as both the anode and cathode in alkaline solution. In addition to the quaternary compound, the construction of trimetallic (Co/Ni/Cu) HP NSs on Ni foam has also been published. Zhang et al. examined the design of CuHP loaded CoNi hybrid nanostructures by an ion exchanging method [125]. The excellent OER catalytic activity (a low overpotential of 370 mV at 50 mA cm^–2^) was mainly attributed to the high-valent cobalt species produced in situ during the electrooxidation process. At the same time, the introduction of cobalt and nickel increased the electrochemically active area and provided more effective active sites for OER. The synergy of the three metals and the combination with the nickel foam matrix improved the conductivity of the electrode.

### Mixed Chalcogenides

#### Oxysulfide

Numerous efforts have been made to develop oxysulfides (OS) to achieve high performance in electrocatalysis [52, 128, 129]. Oxygen anions are typically hard (non-polarized), and sulfur anions are soft (polarized in sulfides). The O and S derived from the same VIA group of the periodic table are easily mixed to modify the compounds. To date, a wide range of TM compounds with oxysulfides, e.g., (CoNi)O_*x*_S_*y*_, Si-MWs@MoO_*x*_S_*y*_, CoO_0.87_S_0.13_/GN, Ni_*y*_Co_1−*y*_O_*x*_S_*z*_, and Mn(Zn, Ge) oxysulfide [8, 130, 131], have been explored as a catalyst for electrochemical applications such as supercapacitors [132], PEC [130], Zn-air batteries [133], PEMWE [134] and ORR [135].

Subsequently, Suntivich's group investigated a partial S anionic substitution in CoO NPs to increase the H_2_ evolution [50]. The excellent electrocatalytic activity of CoO_*x*_S_*y*_ was attributed to the exchange of anion S^2–^ and O^2–^ in Co-oxysulfide NPs, which decreased the intermediate energy during HER. However, exchanging more S atoms would increase the H* intermediate energy and decrease HER kinetics, resulting in a lower performance. In another example, based on TM-oxysulfide from anion exchange strategy, Cai et al. prepared amorphous CoO_0.6_S_4.6_ porous nanocubes (PNCs) from corresponding CoFe Prussian blue analogue (PBA) as a precursor [48]. The local disordered structure was generated due to the insertion of O atoms around the Co center, which was evidenced by significantly decreased intensity beyond the first shell peaks. Besides, the Co atom is surrounded by 4.6 S atoms and 0.6 O atoms with abundant Co–S dangling bonds. These Co–S dangling bonds and oxygen incorporation into the CoS_*x*_ host can greatly enhance O* adsorption, thereby significantly improving the activity on a single site. More impressively, the obtained CoO_0.6_S_4.6_ PNCs showed excellent performance for water oxidation with a low onset potential of 1.52 V (1 M KOH), 1.50 V (0.1 M PBS) to achieve a *j* of 10 mA cm^–2^.

**Fig. 9:**
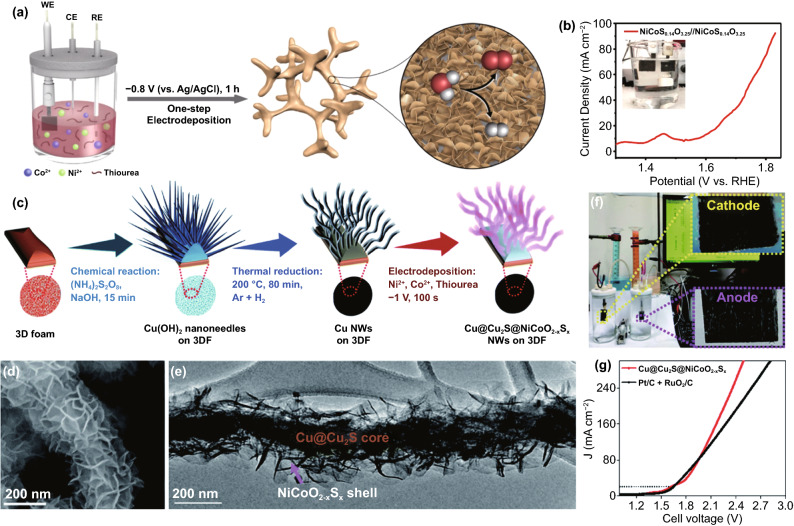
**a** Schematic illustration of the synthesis of 3D NiCoSxOy nanosheet arrays on Ni foam, and the application as OER and HER catalysts for overall water-splitting, **b** two electrode OER polarization curves (without iR compensation) of NiCoS_0.14_O_3.25_ NSs/NF//NiCoS_0.14_O_3.25_ NSs/NF at a scan rate of 1 mV s^−1^. Inset: A photograph of the all Ni–Co nanosheets arrays-based electrolyzer. Reprinted with permission from Ref. [136]. Copyright Year 2018. Elsevier. **c** Schematic illustration of the Cu@Cu_2_S@NiCoO_2−*x*_S_*x*_ NW fabrication, **d** FE-SEM and **e** TEM image images of Cu@Cu_2_S@NiCoO_2−*x*_S_*x*_ NWs on 3DF, **f** the fabrication of an electrolyzer based on cathodic and anodic Cu@Cu_2_S@NiCoO_2−*x*_S_*x*_ NW electrodes, **g** LSV measurement for the overall water-splitting performance of the electrolyzer devices based on Cu@Cu_2_S@NiCoO_2−*x*_S_*x*_ NW electrodes and (Pt/C + RuO_2_/C) electrodes. Reprinted with permission from Ref. [137]. Copyright Year 2020. Royal Society of Chemistry

Self-supporting structures own the advantages of high current density and simple preparation. Li et al. prepared Ni foam supported 3D NiCo sulfoxide (NiCoS_*x*_O_*y*_) nanosheets through electrodeposition on NF with Ni, Co, S precursors (Fig. 9a) [136]. As a result, a cell voltage of 1.64 V was required to attain a 20 mA cm^–2^ current density (Fig. 9b). The electronegativity of the S ligand was comparable to OH species in alkaline electrolyte, which made the OH species attract electrons intensively, inhibiting the transformation electron from the M–S to M–O bond, which resulted in a shorter and more stable M–S bond in NiCoS_0.14_O_3.25_ than the other TM sulfides. On the other hand, the nanosheet arrays constructed from O^2–^ and large S^2–^ anions with Ni and Co cations at different valence states could help diverse active sites achieve lower activation potential.

NiFe oxysulfide is also very active as a bifunctional catalyst. For example, Liu et al. reported the in situ grown fullerene-like nickel oxysulfide hollow nanospheres on 3D Ni foam by a solvothermal method [138]. The as-prepared catalyst showed a low overpotential of 0.29 V and 0.14 V at 10 mA cm^–2^ for OER and HER, respectively. Similarly, Li et al. fabricated NiFe oxysulfide (NiFeS-2) by the vulcanization treatment of NiFe layered double hydroxide (LDH) precursor using thioacetamide [59]. The catalyst exhibited superior OER performance (*η*_10_ = 0.286 V) because the polarized S and non-polarized O anions equally adjusted the electronic configuration of the active sites in the catalyst. For overall water-splitting, a *j* of 10 mA cm^–2^ was achieved at 1.64 V in N_2_ saturated 1 M KOH solution with NiFeS-2/NF electrode served as anode and cathode. Furthermore, Tran et al. designed a novel catalyst derived from a porous interconnected network of nickel cobalt oxysulfide interfacial assembled Cu@Cu_2_S nanowires (NWs) via a three-step process (Fig. 9c–e) [137]. In 1.0 M KOH medium, the catalyst only required an overpotential of 203 mV to achieve a current response of 295 mV to reach 50 mA cm^−2^ for the OER and 20 mA cm^−2^ for the HER. Besides, a developed electrolyzer enabled a small cell voltage of 1.61 V at 20 mA cm^−2^ without performance decay upon long-term operation (Fig. 9f–g).

#### Phosphosulfides

Nowadays, TM phosphosulfides (TMPS) exhibit exceptional catalytic performance. The electrocatalytic activity of S completely depends on the electron-donation of chalcogen ligands (X_2_^2–^). While the P atoms enhance the electron-donating ability of X_2_^2–^, which encourages the redox reactions of metal atoms [68]. Recently, numerous reports are available in the literature for TM-based phosphosulfides such as MoPS, PdPS, CoPS, FePS_3_, NiPS_3_, and so on [26, 66, 139–147].

In this regard, Kibsgaard et al. developed a wet chemical route to prepare MoPS with sulfidation treatment [148]. The anion-mixed MoPS catalyst demonstrated a low overpotential of 0.064 V at 10 mA cm^–2^ in acidic media for HER (sample supported on Ti foil), which is much better than that of MoP/Ti-based catalyst. Palladium (Pd) with phosphosulfide also possesses excellent electrochemical properties. From the report by Sampath's group, PdPS/rGO composites exhibited excellent HER activity with a low overpotential of approximately 0.09 V versus RHE and a small Tafel slope of 46 mV dec^−1^ [149]. It is worth mentioning that the overall structure was found to be “layered-type” made up of pentagons joints to result in wrinkled two-dimensional sheets, in which four layers are needed to generate one unit cell. The P and S atoms are estimated to form polyanions of type [S–P–P–S]^4–^. The same group also used few-layered 2D FePS_3_ nanosheets as the composite material for preparing rGo–FePS_3_ composite electrocatalysts [150]. It was proposed that a direct bond between P and S in the FePS_3_ could enhance its HER activity. The reason for the enhanced HER activity was further supported by DFT calculations, which confirmed that the presence of [P_2_S_6_]^4−^ (P and S sites) unit could participate in the adsorption and desorption of the H atom.

In a few reports, flexible carbon fiber (CF) conductive substrates have been used for 2D growth. He's group successfully prepared WP_2*x*_S_2(1−*x*)_ nanoribbons (NRs) on CF substrate through phosphatization and sulfidation reaction of WO_3_ NWs [152]. Surprisingly, electronic perturbation also occurred in WS_2_ after doping of P atoms in the WP_2*x*_S_2(1−*x*)_ which played a prominent role in improving HER activity, stability, and durability.

**Fig. 10:**
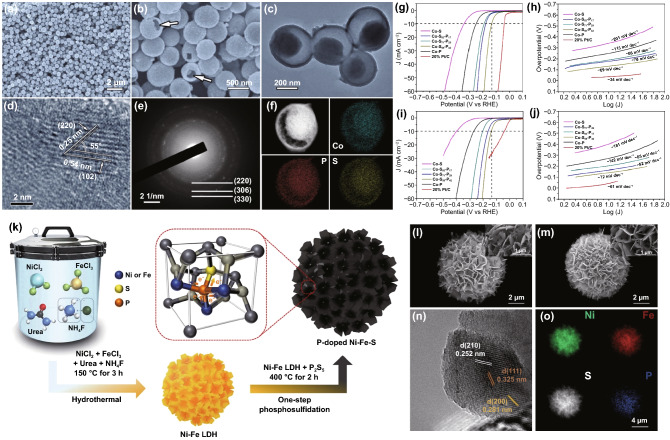
**a** SEM, **b** expanded SEM view. **c** TEM image. **d** High-resolution TEM image. **e** SAED pattern. **f** STEM image and elemental mapping of the Co_0.9_S_0.58_P_0.42_ yolk-shell spheres. **g–j** Electrocatalytic performance of different catalysts for HER in alkaline and acidic electrolytes. **g** Polarization curves and **h** Tafel plots in 0.5 M H_2_SO_4_. **i** Polarization curves and **j** Tafel plots in 1 M KOH. Reprinted with permission from Ref. [64]. Copyright Year 2017. American Chemical Society. **k** Schematic illustration of the preparation of the P-doped Ni–Fe–S microspheres. High-magnification SEM images of **l** Ni–Fe–S, and **m** P-doped Ni–Fe–S, **n** HRTEM image of the P-doped Ni–Fe–S, **o** EDS elemental mapping of the P-doped Ni–Fe–S. Reprinted with permission from Ref. [151] Copyright Year 2021. Elsevier

Pyrite-type transition metal dichalcogenides (TMDs) with non-metal anion incorporation are well-known to be an efficient and high catalytic activity than other TM compounds. As Jin's group reported, ternary pyrite-type cobalt phosphosulfide (CoPS) (NWs, NPs) electrodes show higher HER performance than CoS_2_ NWs and CoSe NPs [68]. Notably, the CoPS electrode exhibited a lower overpotential of 48 mV at the *j* of 10 mA cm^–2^, a Tafel slope of 56 mV dec^–1^, and long-term operational stability (36 h) in acidic solution. As a typical example, our group developed a partial sulfurization/phosphorization strategy to synthesize pyrrhotite-type cobalt mono-phosphosulfide (Co_0.9_P_0.42_S_0.58_) yolk-shell spheres with hexagonal close-packed phase (Fig. 10a–f) [64]. It is worth pointing out that the synergy of non-stoichiometric nature and the adjustable P/S ratio results in the reinforced Co^3+^/Co^2+^ couples and tunable electronic structure. Co^3+^ ions effectively enhance the OER activity due to their higher valence 3d electron orbits and more electron-accepting features than Co^2+^ ions. For HER, it showed a low operating overpotential around 140 mV with small Tafel slopes around 70 mV dec^–1^ in both alkaline and acidic media (Fig. 10g–j). Coupled with the high HER activity of Co_0.9_S_0.58_P_0.42_, the overall water-splitting was demonstrated with a low η_10_ at 1.59 V.

Similarly, layered nickel phosphosulfide is another kind of novel electrocatalyst. For example, Ren's group prepared pure layered NiPS_3_ and NiPS_3_ NSs/Graphene (G) composites as electrocatalysts toward OER [153]. They believed that non-metallic elements such as P and S generally do not directly contribute to OER performance. However, due to their strong electronegativity, they can affect the local electronic structure of Ni, thereby promoting the oxidation of Ni^2+^ to Ni^3+^. Besides, NiP_0.62_S_0.38_/NiOOH heterostructure displayed increased OER performance [65]. For HER, the NiP_0.62_S_0.38_ nanosheets owned a small overpotential of 52 mV at 10 mA cm^–2^, whereas an overpotential of 240 mV was observed at 10 mA cm^–2^ for the OER. It is noteworthy that the electronegative phosphosulfide species moved ΔG_H*_ to the thermally neutral position and achieved the balance of hydrogen adsorption/desorption at the nickel position.

Apart from ternary PS compounds, the HER, OER activities of multicomponent mixed PS-based materials have also been investigated [154]. For example, using P_2_S_5_ as a source, 3D hierarchical flower-like P-doped Ni–Fe disulfide microspheres were prepared by successive hydrothermal and one-step phosphosulfidation (Fig. 10k–o) [151]. These microspheres deliver outstanding OER performance with an overpotential of 264 mV to achieve a *j* of 10 mA cm^−2^ and excellent long-term durability over 50 h with negligible degradation in alkaline media.

#### Sulfoselenide

Transition metal chalcogenides (TMCs) are an extremely attractive material with unique electronic structures and physicochemical properties [31, 103, 155–159]. In particular, the electrocatalytic activity of TMDs has received considerable attention [156, 160, 161]. Controlling the S, selenium (Se) substitution, and varying composition ratios can improve the HER/OER performance [160, 162, 163].

Liu et al. fabricated a ternary cobalt sulfoselenide (CoS_2*x*_Se_2 (1−*x*)_) NW arrays via a two-step process supported on CFs [164]. The unique electronic structure, large surface area, and lower charge transfer resistance of CoS_2*x*_Se_2(1−*x*)_ NWs could augment its HER performance. More impressively, Xu's group replaced S atoms with Se in molybdenum sulfoselenide (MoS_2*x*_Se_2(1−*x*)_) alloys and thus achieved high electrical conductivity with a narrowing bandgap [72]. Also, Se incorporation expanded the interlayer distance (Se atoms have a larger size than S atoms) and modified edge electronic structure, and hence improved the HER performance. The HER activity of ternary MoS_2*x*_Se_2(1−*x*)_ (*η*_10_ = 0.219 V) was better than that of the binary MoS_2_. Similarly, ternary tungsten sulfoselenide (WS_2(1−*x*)_Se_2*x*_) particles were grown on 3D porous NiSe_2_ foam as an efficient HER electrocatalyst [165]. This catalyst demonstrated high conductivity, large specific surface area, and high density of active edge centers, with a *η*_10_ = 88 mV. Except for 3D NiSe_2_ foam, FTO is another ideal conductive substrate for growing TMDs. Hussain et al. deposited MoS_2(1−*x*)_Se_2*x*_ and WS_2(1−*x*)_Se_2*x*_ alloys on FTO by RF magnetron sputtering of Mo, W target (Ar atmosphere) followed by sulfurization and selenization processes [166]. Finally, the resulting MoS_2(1−*x*)_Se_2*x*_ and WS_2(1−*x*)_Se_2*x*_ films performed an overpotential of 0.141 and 0.167 V at a *j* of 10 mA cm^−2^ for HER.

**Fig. 11:**
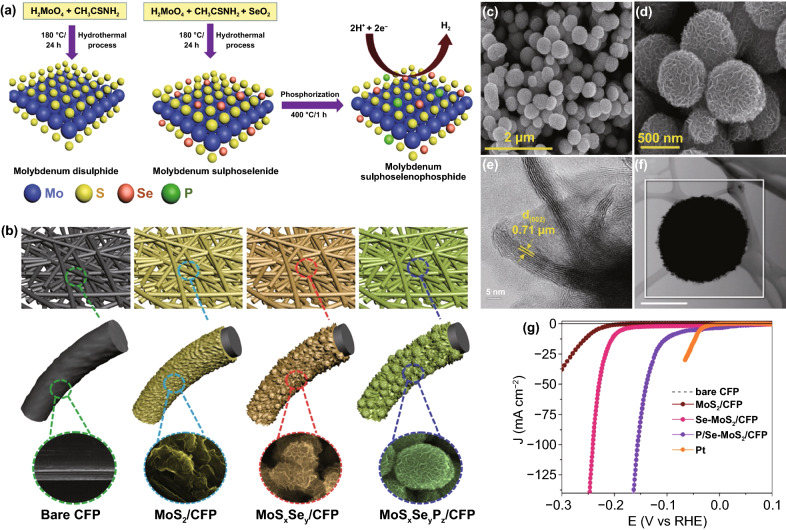
**a** Schematic illustration of the fabrication of the MoS_2_, MoS_*x*_Se_*y*_, and MoS_*x*_Se_*y*_P_*z*_ on CFP by facile hydrothermal and phosphorization process. **b** Schematic diagram to illustrate the structural change of CFP, MoS_2_, MoS_*x*_Se_*y*_, and MoS_*x*_Se_*y*_P_*z*_ based on anion incorporation. **c–f** FESEM (P/Se–MoS_2_ spheroids distributed on CFP), HRTEM and STEM images of P/Se–MoS_2_ **g** Polarization curves of as-prepared various catalysts in comparison with commercial Pt and bare CFP. Reprinted with permission from Ref. [158]. Copyright Year 2018, Wiley–VCH Verlag GmbH & Co. KGaA, Weinheim

Except for binary anion mixing, recently, Yi et al. reported the molybdenum sulfoselenophosphide (MoS_*x*_Se_*y*_P_*z*_) catalyst synthesized through a scalable hydrothermal method followed by the phosphorization process (Fig. 11a, b) [158]. When phosphorus was doped, Se–MoS_2_ exposed uniformly distributed microspheres covered by stretched nanoflakes (Fig. 11c–f). These nanoflakes with prominent edges on the circumference of the sphere can be attributed to the numerous edge sites, which is in favor of HER activity enhancement on P/Se–MoS_2_ (Fig. 11g). As a result, this catalyst exhibited high HER catalytic activity (*η*_10_ = 93 mV) and exceptional stability in an acidic medium. The substitution of Se by P significantly changes the morphology of the catalyst, and these p atoms are not only the basis for proton capture but also provide superior activity for H_2_ dissociation. To further explore different compositions of both cations and anions on the performance of HER, the high-temperature solution method has been used to develop a series of ternary and quaternary Mo_*x*_W_1−*x*_(S_*y*_Se_1−*y*_)_2_ alloys [167]. The variation of S-to-Se ratio had a certain impact on bond strength between TMD and H atom intermediates, and the enhanced performance of MoSSe relative to MoSe_2_ or MoS_2_ was owing to its optimal ΔG_H*_. As another interesting sulfoselenide example, Smialkowski et al. developed seleno-substituted pentlandites (Fe_4.5_Ni_4.5_S_8−γ_Se_γ_) (γ = 1–8, Se1–Se8) via a high-temperature solid-state method [168]. The catalytic activity of pentlandite was attributed to the distance between different M sites. Only Se1 displayed a reduced overpotential of 172 mV at 10 mA cm^–2^, defeated the benchmark material pentlandite (190 mV), and confirmed the possibility to further tune up Fe/Ni-chalcogenide catalysts via S/Se exchange.

#### Selenotellurides

For the improvement of catalytic activity, the form of solid solutions in different TMCs plays a significant role [169]. Kosmala et al. has reported a comparative analysis of HER performance for ultrathin films of MoTe_2_, MoSe_2_, and their solid solutions on highly oriented pyrolytic graphite [170]. DFT study has confirmed that the chemical function of the reactive 2H phases could be enhanced by including metallic twin boundaries. The catalytic performance of MoSe_0.12_Te_1.79_ was – 0.41 V after the sputtering and MoSe_0.17_Te_1.83_ with the different stoichiometry exhibited an overpotential of – 0.45 V. It is worth pointing out that after sputtering, a little increase in the activity indicated the creation of anion vacancies caused by oxide removal and metal terminating edges exposure were advantageous to the electrochemical activity up to an extent. And the basal plane of these materials provided high intrinsic activity. The MoTe_2_ was more active than pure MoSe_2_, and the higher fraction of tellurium (Te) (or lower Se) in the selenotellurides constituted better performance.

#### Hydroxy (Sulfates, Sulfides)

**Fig. 12:**
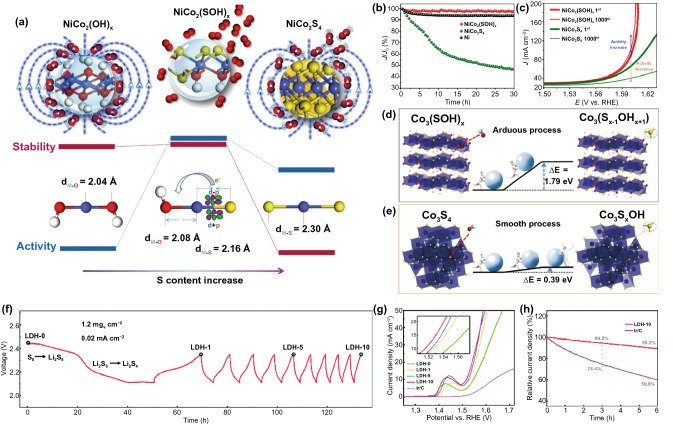
**a** Ultimate unity of OER performance of NiCo_2_(S_*x*_OH_2−*x*_)_*y*_. Here, Co, S, O, and H atoms are shown in blue, yellow, red and white, respectively, **b** Chronoamperometric curves of Ni, NiCo_2_(SOH)_*x*_ and NiCo_2_S_4_ at a current density of 100 mA cm^−2^, **c** first and 1000th polarization curves of NiCo_2_(SOH)_*x*_ and NiCo_2_S_4_. **d, e** Total energy change during the desulfuration process of S^2–^ in Co_3_S_4_ (upper) and Co_3_(SOH)_*x*_ (bottom) replaced by OH^–^. The non-spontaneous process of Co_3_(SOH)_*x*_ with a more positive energy change discloses a more thermodynamic stable structure of Co_3_(SOH)_*x*_ compared to Co_3_S_4_. Reprinted with permission from Ref. [61]. Copyright Year 2017, American Chemical Society. **f** Electrochemical anionic regulation process. Galvanostatic discharge/charge curves of the Li–S cell reactor, **g** 95% iR-compensated LSV curves of the LDH-x and Ir/C electrocatalysts at a scan rate of 10.0 mV s^−1^ and the rotation rate of 1600 rpm in O_2_-saturated 0.10 M KOH electrolyte, **h** Chronoamperometric response of the LDH-10 and Ir/C electrocatalysts for stability evaluation. Reprinted with permission from Ref. [172]. Copyright Year 2020, Royal Society of Chemistry

Like other ternary/quaternary TMCs materials, TM*-*Hydroxy (sulfate, sulfides) are extensively employed in heterogeneous catalysis due to their high activity and robust stability [171]. As an interesting example, the S ligand was incorporated into the stable structure of layered hydroxides to form NiCo_2_(S_*x*_OH_2−*x*_)_*y*_ with a single-phase and homogeneous composition, which inherited the superior stability from the conventional transition metal hydroxides and excellent activity from the conventional transition metal sulfides and even exceeds the predecessors (Fig. 12a) [61]. As an OER catalyst, this optimized NiCo_2_(SOH)_*x*_ exhibited a lower overpotential of 0.29 V to reach a *j* of 10 mA cm^–2^ and a Tafel slope of 47 mV dec^–1^ in 1.0 M NaOH electrolyte. Impressively, the catalyst also showed excellent durability with no decay for OER at large *j* of 100 mA cm^–2^ with 30 h continuous operation (Fig. 12b, c). Furthermore, the DFT calculations revealed that the synergetic effect of OH and S ligands on the surface of NiCo_2_(SOH)_*x*_ were able to tune the electronic structure and their chemical environment around the metal active centers. This resulted in optimal binding energies of the OER intermediates (*OH, *O, and *OOH) and enhanced the binding energy between M and S anion, rendering for intrinsically improved activity and durability respectively (Fig. 12d, e).

Very recently, Zhao et al. made a systematic investigation on NiFe hydroxysulfide-*x* (LDH-*x*. x represents the cyclic number) with oxygen–sulfur hetero-anionic structure served as outstanding OER catalysts [172]. An electrochemical reaction assisted by an anionic regulation strategy was proposed for the precise construction of advanced electrocatalysts (Fig. 12f). Specifically, by precisely regulating the anion, the LDH-10 showed greatly enhanced performance with a low overpotential of 0.286 V at 10 mA cm^–2^ (Fig. 12g). Besides, the LDH-10 electrocatalyst also exhibited remarkable stability, maintaining 89.2% of the initial OER current density after a 6.0 h potentiostatic test (Fig. 12h).

#### Phosphoselenide

The substitution of Se into phosphides (PSe) would be the next significant step toward outstanding electrochemical properties. The Se doping enhances the electron transfer from interior phosphides and provides more active sites [173, 174]. Although enormous research have been carried out in the field of mixed chalcogenides, there is still room for improvement in the electrochemical properties and the construction of TM binary/ternary hybrid phosphoselenide at a maximum level.

**Fig. 13:**
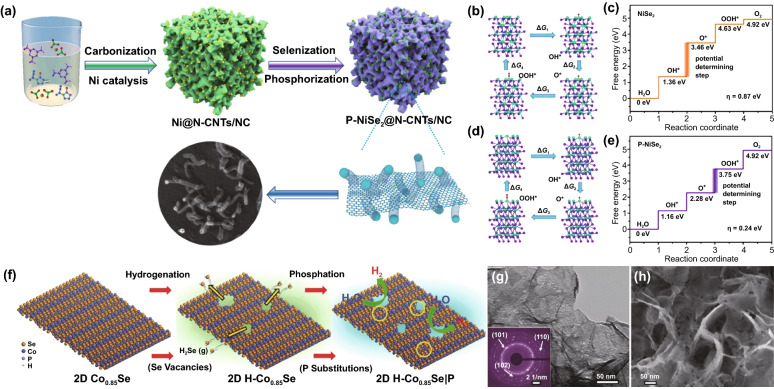
**a** Schematic illustration for the preparation procedure of P–NiSe_2_@N–CNTs/NC, **b** Schematic illustration for the four elementary steps during oxygen generation process on NiSe_2_, **c** Calculated free-energy diagram for the OER paths on NiSe_2_ at zero potential, **d** Schematic illustration for the four elementary steps during oxygen generation process on P–NiSe_2_ in 1 M KOH, **e** Calculated free-energy diagram for the OER paths on and P–NiSe_2_ at zero potential. Ni, Se, P, O, and H atoms are represented by green, purple, pink, red and white spheres, respectively. Reprinted with permission from Ref. [73]. Copyright Year 2021, Elsevier. **f** Schematic illustration for the synthesis process of H–Co_0.85_Se|P, **g** TEM image of H–Co_0.85_Se|P, **h** FESEM image of H–Co_0.85_Se|P. Reprinted with permission from Ref. [176] Copyright Year 2017, Wiley–VCH Verlag GmbH & Co. KGaA, Weinheim

For example, Yu et al. prepared the P–NiSe_2_@N-CNTs/NC hybrid catalyst via a two-step method (Fig. 13a), which delivered low overpotentials of 95 and 306 mV at 10 mA cm^−2^ for HER and OER in alkaline media, respectively [73]. DFT calculations revealed that the electron density surrounding Ni atoms was reduced while the charges accumulated around Se due to P doping, respectively, which in turn reduced the energy barriers for both water dissociation and intermediates adsorption for both HER and OER (Fig. 13b–e). A cell assembled by P-NiSe_2_@N-CNTs/NC hybrid catalyst-based anode and cathode performed a low applied voltage of 1.609 V to reach 10 mA cm^−2^ with outstanding long-term stability. Besides, He et al. demonstrated that cobalt phosphoselenide (CoP_2*x*_Se_2(1−*x*)_) was an efficient electrocatalyst with overpotentials of 70 mV and 98 mV for HER in acidic and alkaline media, respectively, and 0.29 V for OER in alkaline media to achieve 10 mA cm^–2^ [175]. The excellent HER activity of CoP_2*x*_Se_2(1−*x*)_ can be attributed to the substitution of P atoms into CoSe_2_ to form a unique electronic structure.

Besides, Hou et al. successfully prepared porous cobalt phosphoselenide nanosheets (H–Co_0.85_SeP) by the combined hydrogenation and phosphation method (Fig. 13f) [176]. Figure 13g, h shows the HRTEM and FESEM images of ternary H–Co_0.85_SeP nanosheets. The H–Co_0.85_SeP NSs became higher porous, which can be attributed to the structural disorder of NSs through the phosphorization reaction. It should be mentioned that the presence of selenium vacancies and the subsequent phosphorus displacement of selenium atoms around the vacancies favorably changed the electronic structure of cobalt selenide, ensuring rapid charge transfer and the best energy barrier for hydrogen desorption, thereby promoting proton dynamics. The H–Co_0.85_SeP catalyst exhibited superior HER activity with current densities of 10 and 20 mA cm^–2^ at an overpotential of 0.15 and 0.18 V. Finally, using ternary electrode as both anode and cathode, the overall-water-splitting with 10 mA cm^−2^ at a low voltage of 1.64 V was achieved, which is better than that of the Ir/C–Pt/C couple.

### (Oxy)hydroxides

Considerable efforts have been constructed in the field of TM-(oxy)hydroxides (OOH) to produce highly efficient and stable electrocatalysts [177–184]. TM–OOH are constructed by layered-stacking TMO_6_ octahedrons with protons sandwiching between layers, and the different intercalating species can alter the layer spacing. Especially, Ni, Fe, Co, and Mn-based (oxy)hydroxides have been extensively studied as highly efficient catalysts for water-splitting owing to their diversity, numerous active sites in alkaline medium, potential stability, and low cost [131, 185–193].

Shen et al. synthesized stainless steel (SS) nanocone array coated with layer NiFe oxides/(oxy)hydroxides with more active edges, which eventually contributed to the promotion of OER activity (*η*_10_ = 0.232 V and 340 h stability (20 mA cm^–2^)) [194]. Similarly, Sun's group recently fabricated hierarchical layered structured FeNiO_*x*_H_*y*_ as an efficient OER electrocatalyst [195]. The electrocatalyst was synthesized on Ni foam via simple electrodeposition through surface modification with pretreatment by phosphoric acid. In alkaline solution (1 M KOH), to reach a *j* of 10 and 50 mA cm^–2^, FeNiO_*x*_H_*y*_/NF (Phosphoric acid treatment) only required overpotential of 0.206 and 0.234 V, whereas the values are 0.253 and 0.279 V for conventionally electrodeposited FeNiO_*x*_H_*y*_/NF. They claimed that the enhanced intrinsic activity was caused by the anion exchange of phosphate to (oxy)hydroxide.

To gain deeper insights into the role of metal dopants in enhancing (oxy)hydroxides’ OER activity, Ir-doped Ni(OH)_2_ nanosheets were fabricated through a combination of hydrothermal assembly and liquid exfoliation, with the nanosheets transforming to Ir-doped NiOOH during OER and offering superior activity relative to pristine Ni(OH)_2_ nanosheets or a commercial IrO_2_ catalyst [196]. The DFT calculations with Hubbard U correction indicated that Ir doping increased the conductivity of β-NiOOH(001) and activated the oxygen site containing 3 Ni atoms (Ni_3_ site). Boettcher et al. investigated emphasized the significant influence of iron ion OER catalyst activity [186]. Under OER conditions, thermodynamics seems more inclined to hydrated phases, such as (oxy)hydroxides or hydrous oxides, rather than crystalline oxides. Iron impurities are ubiquitous in neutral and alkaline electrolytes, which can significantly improve the activity of Ni and Co-based OER catalysts. The active center may be Fe, and the host provides a conductive framework with a high specific surface area, which is chemically stable and further activates the Fe center.

Alternatively, TM–OOH porous nanostructure arrays on carbon fiber cloth (CFC) are outstanding OER catalysts. For example, Fe-substituted CoOOH (Fe_*x*_Co_1–*x*_OOH, 0 ≤ x ≥ 0.33) with uniformly dispersed porous nanosheet arrays on CFC was also reported [197]. Undoubtedly, Fe_0.33_Co_0.67_OOH/CFC exhibited exceptional OER catalytic performance with a low overpotential of 0.26 V at 10 mA cm^–2^. Besides, Fe-incorporated CoNi (oxy)hydroxide (Fe–CoNi–OH) nanosheet-assembled nanorod arrays were fabricated [198]. Fe-incorporated effection exposed more active sites, facilitated the mass transfer, modified CoNi (oxy)hydroxide (CoNi–OH)'s electron structure, and enhanced its electronic conductivity, thus promoting the intrinsic OER activity. Consequently, the Fe–CoNi–OH possessed excellent OER activity with low overpotentials of 210, 248, 304, and 349 mV to achieve current densities of 10, 100, 500, and 1000 mA cm^−2^, respectively.

**Fig. 14:**
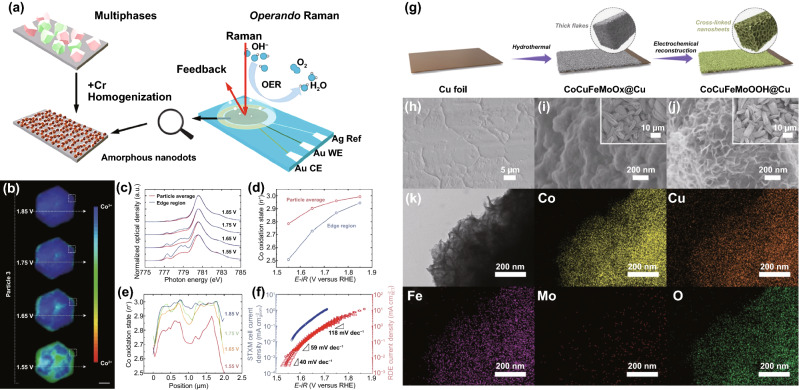
**a** Scheme of the amorphization by introducing Cr and operando Raman testing during OER. Reprinted with permission from Ref. [199]. Copyright Year 2020. American Chemical Society. **b** Phase maps of a β-Co(OH)_2_ particle at applied voltages in different OER Tafel regions. Scale bar, 500 nm, **c** Co LIII-edge STXM–XAS spectra of the particle average and the boxed edge region in a, showing a reduced Co oxidation state at the edge of the particles, **d** Corresponding Co oxidation state for the full particle average and edge region from the Co LIII-edge XAS spectra in **c**, **e** Co oxidation state line scans for the lines shown in **b**, **f** OER Tafel data taken in the STXM cell and in a macroscopic RDE cell at a scan rate of 10 mV s^−1^. Reprinted with permission from Ref. [200]. Copyright Year 2021. Springer Nature. **g** Schematic illustration of the synthesis of CoCuFeMoOOH@Cu, **h** SEM image of bare Cu foil as the substrate, **i** SEM images of CoCuFeMoOx and **j** CoCuFeMoOOH, **k** EDS elemental mapping images of Co, Cu, Fe, Mo, and O for CoCuFeMoOOH. Reprinted with permission from Ref. [201]. Copyright Year 2021. Wiley–VCH Verlag GmbH & Co. KGaA, Weinheim

Recently, operando technique has become a powerful method to study the real-time catalytic state of OER reaction. For example, Zhao and coworkers revealed that the crystalline multiphase NiFe and CoFe oxides/hydroxides could be efficiently transformed into homogeneous amorphous nanodots through the introduction of Cr [199]. Through operando electrochemical Raman spectroscopy, the impact of Cr on the NiFe and CoFe catalysts for OER kinetics was systematically studied (Fig. 14a). For the CoFe and CoFeCr systems, the introduction of Cr only disturbed the lattice crystallization. For the NiFeCr compound, Cr could promote the generation of a more active β-NiOOH phase than that of the NiFe composite during water oxidation. Besides, through a suite of correlative operando scanning probe and X-ray microscopy techniques, a link between the oxygen evolution activity and the local operational chemical, physical, and electronic nanoscale structure of single-crystalline β-Co(OH)_2_ platelet particles was established [200]. The interlayer water and protons de-intercalate to form contracted β-CoOOH particles that contain Co^3+^ species upon increasing the voltage to drive oxygen evolution. The observed Tafel behavior is correlated with the local concentration of Co^3+^ at these reactive edge sites, demonstrating the link between bulk ion insertion and surface catalytic activity (Fig. 14b–f).

Furthermore, a design combining ultrathin, amorphous, and alloyed oxyhydroxide has also been paid great attention. For example, Yang's group prepared ultrathin amorphous cobalt–vanadium bimetal oxyhydroxide (CoV-UAH) as a promising electrocatalyst for OER [202]. The CoV-UAH performed more structural flexibility than bulky highly crystalline CoV-C and exhibited an outstanding OER catalytic activity with a smaller Tafel slope (44 mV dec^–1^) and abundant defects.

Due to customizable electrochemical performance and excellent reactivity, high-entropy materials have become a new generation of electrocatalysts for water-splitting. A novel high-entropy Co–Cu–Fe–Mo (oxy)hydroxide electrocatalysts were fabricated by a new low-temperature electrochemical reconstruction method (Fig. 14g–k) [201]. These as-prepared quaternary metallic (oxy)hydroxides demonstrated a low overpotential of 199 mV at *j* = 10 mA cm^−2^, a Tafel slope of 48.8 mV dec^−1^, and excellent stability without decay over 72 h in 1 M KOH. The performance enhancement mechanism is due to the usefulness of high-entropy design and the tremendous synergistic effect by incorporating four elements.

Moreover, many discussions on lattice oxygen boost OER activity in oxyhydroxide have been reported [49, 203]. The OER catalyst operated with LOM can break the linear scale relationship in the traditional adsorbate evolution mechanism (AEM) [203]. The catalytic species in various OER catalysts are essentially transition metal oxyhydroxides, and their low dimensional layered structure is conducive to the direct formation of O–O bonds. Huang et al. reported CoOOH OER catalysts doped by low-valence and catalytically inactive Zn^2+^ [49]. They proposed that the OER mechanism depended on the amount of Zn^2+^ in the catalyst. Specifically, only when two adjacent oxidized oxygens could hybridize their oxygen holes without significantly sacrificing metal–oxygen hybridization could OER be carried out on metal oxyhydroxides through the LOM pathway. The prepared Zn_0.2_Co_0.8_OOH displayed the best catalytic activity, showing a low OER overpotential of 235 mV (10 mA cm^−2^_disk_) in 1 M KOH. Their new insights into LOM provide a reference for the development of efficient oxyhydroxides catalysts.

### Mixed Borides/Borates

Among kinds of cost-effective electrocatalytic water-splitting catalysts, transition metal borides/borates (TMB) have caused a great sensation in the past decade. Particularly, the research on the influence of metal-B bonds in TMB on electron transfer is more intense. For example, B plays a sacrificial effect by providing electrons in many amorphous Co and Ni-based borides. The reverse electron transfer from B to the metal causes the enrichment of electrons at the metal site, preventing the metal site's oxidation and improving its stability while promoting electrochemical reactions [204]. In addition, Ai et al. demonstrated that the strong hybridization of Ru *d*_*xz*, *yz*_ orbital and B *sp* orbital would cause the downward shift of *d*-band center in RuB, and the adequate adjustment of the electronic structure made RuB a promising and efficient HER catalyst [205]. The study by Ma et al. exhibited that highly electronegative B atoms could reduce the density of states of Ni-3d orbitals near the Fermi level, thereby optimizing the binding energy of OOH* [206].

Recently, the incorporation of phosphorous (P) has become an efficient strategy to improve the catalytic performance of TMB. In these mixed borides, P plays a vital role in changing the electronic structure and enhancing their activity. Ma et al. displayed a nickel–borate–phosphate nanoarray (Ni–Bi-pi/CC) prepared by topological transformation, which owned a high catalytic activity of only 440 mV overpotential (10 mA cm^–2^) in 0.1 M potassium borate (KBi) solution [208]. This electrode also showed 100% Faraday oxygen evolution efficiency and long-term electrochemical durability. Similarly, an in situ derived iron phosphate–iron borate nanosheet array (Fe–Pi–Bi/CC) has also been reported, which only required an overpotential of 434 mV to drive a geometric catalytic current density of 10 mA cm^–2^ and maintained its activity for at least 20 h within 0.1 M KBi [209].

**Fig. 15:**
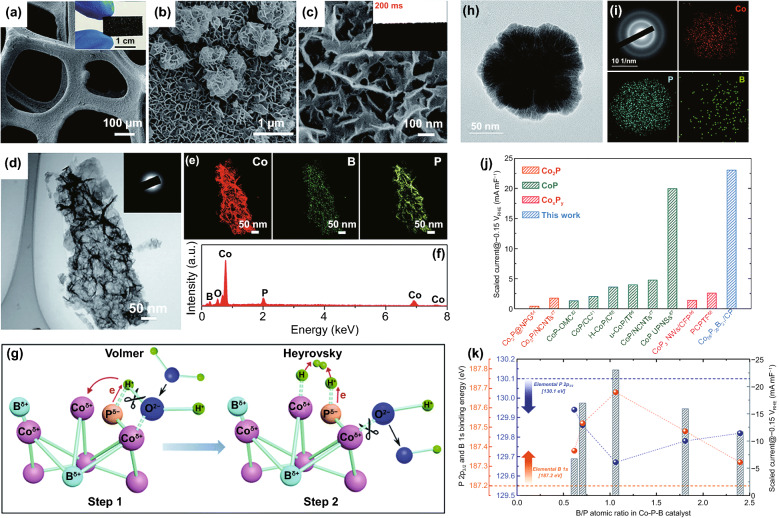
**a** Low-magnification and **b**, **c** high-magnification SEM images of synthesized Co–B–P/NF, **d** TEM image and SAED pattern (inset), **e** elemental mapping and **f** the corresponding EDS spectrum of Co–B–P sheets scraped off from the Ni foam substrate, **g** Schematic HER electrocatalysis on Co–B–P with a synergistic effect of Co, B and P. Reprinted with permission from Ref. [74]. Copyright Year 2018. Royal Society of Chemistry. **h** TEM image, **i** SAED patterns and elemental mapping images of Co_59_P_20_B_21_/CP catalyst, **j** Comparison of HER performance in 0.5 M H_2_SO_4_ with other CoP_*x*_ electrocatalysts reported in literature, **k** Scaled current of Co–P–B catalyst corresponding to binding energy of P 2*p*_3/2_ (navy) and B 1*s* (orange) with atomic ratio of catalyst. Reprinted with permission from Ref. [207]. Copyright Year 2018. Royal Society of Chemistry

In a typical study, through a scalable one-step chemical deposition method, the Co–B–P micro/nanostructure was directly synthesized on a foamed nickel substrate, showing a porous and interconnected thin nanosheet configuration (Fig. 15a–d) [74]. As shown in Fig. 15e–f, the B and P elements are uniformly distributed. As a result, Co_2.90_B_0.73_P_0.27_ nanosheets exhibited excellent HER catalytic performance with an initial overpotential of 12 mV and a Tafel slope of 42.1 mV dec^−1^ in alkaline media. Besides, this self-supporting monolithic electrode could maintain a high current density of 1000 mA cm^–2^ and extend polarization for more than 20 h. The reasonable reaction mechanism of B and P synergistically enhancing the catalytic performance was discussed in detail, as shown in Fig. 15g. In step 1, the interaction between Co^δ+^ and O^2–^, P^δ–^, and H^+^ could enhance the adsorption of H_2_O, weaken the HO–H bonds, and promote the effective dissociation of H_2_O. In step 2, OH^−^ produced by H_2_O dissociation combined with Co^δ+^ around P, and H was transferred to nearby Co^δ+^. For Co–P, H combined strongly with Co^δ+^, which was unfavorable for H_2_ evolution. By contrast, in the ternary Co–B–P, the electron transfer from B to Co could inhibit the oxidation of the Co-active site, and the chemical bond was optimized, thereby increasing HER activity. In another study, the Co–P–B catalyst was directly electrodeposited on carbon paper (CP) [207]. The Co_59_P_20_B_21_/CP particle showed a size in the range of 100–200 nm (Fig. 15h, i) and displayed higher intrinsic activity than other CoP_*x*_ HER catalysts reported in previous literature (Fig. 15j). Interestingly, the intrinsic activity of HER and the B/P ratio in the Co–P–B catalysts presented a volcanic function relationship (Fig. 15k). The electron transfer between the three elements was an essential factor to improve the inherent activity of HER. The highest activity was obtained when the B/P ratio was around 1, and the electron transfer was maximized between the three elements. In addition to the Ni/Co-based system, Ma et al. recently designed a B, P co-doped ternary NiVFe layered double hydroxide nanosheet (NiVFe–B–P LDHs@NF) [210]. The results show that B, P co-doping promoted the formation of defects and amorphous regions, increasing the active sites and active surface area.

## Summary and Future Perspective

In this review, we have summarized the recent research advances of non-precious metal electrocatalysts for oxygen evolution reaction, hydrogen evolution reaction, and bifunctional (HER/OER) activity with particular attention on anion mixed strategy. Tables 1, 2 and 3 summarize the various types of mixed anions, substrate, electrolyte, and electrochemical performance of anion-mixed electrocatalysts based on water-splitting activity. The incorporation/replacement of anions like O, S, N, P, F, Se, Te, B, and OH to form OS, ON, OS, OSe, PO, OOH, SSe, SeTe, OHS, PS, and BP into TMs have been demonstrated as a promising strategy in regulating/adjusting the electronic structure of materials, creating defects and vacancies that served as active sites. Finally, it is concluded that anion-mixed electrocatalyst is one of the fascinating and reliable methods to achieve cost-efficient and high-performance electrocatalysts for water hydrolyzers.

**Table 1:** Comparison of the OER and HER performance of typical mixed nitrides, halides, and phosphides-based electrocatalysts

Catalyst	Mixed anions	Electrolyte	Substrate	Overpotential (mV) HER (10 mA cm^−2^)	Overpotential (mV) OER (10 mA cm^−2^)	Tafel slope (mVdec^−1^)	Stability	Refs.
WON@NC NAs/CC	ON	0.5 M H_2_SO_4_ 1.0 M PBS 1.0 M KOH	Carbon cloth	106 152 130	– – –	65	30 h	[211]
CoMnON	ON	0.1 M KOH	Glassy carbon	–	470	79	10,000 cycles	[212]
CoON PNS-400	ON	1 M KOH	Graphite disk	–	230	48	20 h	[84]
TiNO films-25 mTorr	ON	1 M KOH	TiNO thin films	–	290	57	12 h	[85]
CVN/CC	ON	1 M KOH	Carbon cloth	118	263	64 & 73	100 h	[90]
FeCoNi	ON	1 M KOH	Glassy carbon	–	291	64	1000 cycles	[86]
FeNi	ON	1 M KOH	Glassy carbon	–	295	66	1000 cycles	[91]
Fe–WCN (800 °C)	CN	0.1 M H_2_SO_4_ 0.5 M Na_2_SO_4_	Glassy carbon	100 120	–	47	3000 1000 cycles	[213]
HP-CoNC-L	CN	1 M KOH	Glassy carbon	–	300	85	12 h	[214]
WCN	CN	0.5 M H_2_SO_4_ 1 M KOH	Glassy carbon	128 138	–	65	50 h	[92]
N–W_2_C/WC	CN	1 M KOH	Ni foam	63	–	73	60 h	[93]
N–Mo_2_C	CN	0.5 M H_2_SO_4_ 1 M KOH	Glassy carbon	60 48	–	31 34	40 h 40 h	[96]
Boron carbonitride (BCN)	CN	1 M KOH	Carbon Paper	50	170 (onset)	61.3 & 65.2	48 h	[98]
NiFeOF	OF	1 M NaOH	Stainless steel	253	295	96 & 38	10 ks	[29]
IFONF	OF	1 M KOH	Fe foil	20 (onset)	260	31 & 45	30,000 s & 100,000 s	[31]
Co_3_Sb_4_O_6_F_6_	OF	1 M KOH	Glassy carbon	–	443	84	64 h	[107]
NiCoFO	OF	1 M KOH	Ni foam	–	350	23	10 h	[101]
NiFe_2_F_4.4_O_1.8 with CoS*x*_	OF	1 M KOH	Carbon Paper	100	270	40–60	270 h	[100]
CFO-RH400	OF	1 M KOH	Glassy carbon	–	230	68	10,000 cycles	[106]
La_0.5_Ba_0.25_S_r0.25_CoO_2.9–δ_F_0.1_ (LBSCOF)	OF	1 M KOH	Glassy Carbon	256 (100 mA cm^−2^)	518 (100 mA cm^−2^)	44 & 113	700 min	[104]
CoMoOF/GF	OF	0.5 M H_2_SO_4_ 1 M KOH	Graphite felt	94 79	–	60.2 43.3	100 h	[108]
FeVNbTiZrOF	OH–F	1 M KOH	Carbon fiber paper	–	348 ± 2	110.3 ± 0.1	50 h	[113]
Co(OH)F	OH–F	1 M KOH	Glassy carbon	–	313	52	10 h	[60]
NiFe–OH–F	OH–F	1 M KOH	Ni foam	91	–	–	12 h	[109]
NiFe–OH–F–SR	OH–F	1 M KOH	Ni foam	–	176	23	165 h	[110]
(NiFe–PBA)-F	OH–F	1 M KOH	Ni foam	–	190	57	50 h	[112]
F-activated Ni(Fe)O_*x*_H_*y*_	OH–F	1 M KOH	Ni foam	–	218 ± 5	31 ± 4	50 h	[111]
Fe (Ni/Co) (PO_4_)(OH)	(PO_4_) (OH)	1 M KOH	Ni foam	145&160	220&235	43&54 51&57	24 h	[127]
CoFePO	PO	1 M KOH	Ni foam	88	275	52 & 38	100 h	[116]
Co_3_FeP_*x*_O	PO	1 M KOH	MMOF	–	291	85	18 h	[8]
Activated Mn–Co	PO	1 M KOH	Glassy carbon	–	320	52	8 h	[118]
M_*x*_O@M_*x*_P/PNCF	PO	1 M KOH	NiCo foam	256 (500 mA cm^−2^) 343 (1000 mA cm^−2^)	–	40	100 h	[120]
Ni–Zn NSs	PO	1 M KOH	Glassy carbon	–	290	40	30 h	[122]
3D-1D Co–ZnP /CNTs	PO	1 M KOH	Glassy carbon	–	281	41	24 h	[123]
CoFeOP	PO	1 M KOH	Glassy carbon	180	280	53&62	10 h	[119]
FeCoOP	PO	1 M KOH	Carbon fiber paper	–	269	31	100 h	[215]
H–NiFeOP	PO	1 M KOH		–	253	59	20 h	[121]
NiMnOP	PO	1 M KOH	Ni foam or carbon cloth	189	191	29	36 h	[115]
Sn–Fe–HP	OH–P	1 M KOH	Glassy carbon	–	359	81	13 h	[126]
Fe_2.95_(PO_4_)_2_(OH)_2_	OH–P	1 M KOH 1 M H_3_PO_4_	Stainless steel metal plates	– 165.7	281 –	46.48 85.54	12 h 12 h	[124]
CoNi–CuHP/NF	OH–P	1 M KOH	Ni foam	–	370 (50 mA/cm^2^)	88	45 h	[125]

**Table 2:** Comparison of the OER and HER performance of typical mixed chalcogenides (oxysulfides, phosphosulfides)-based electrocatalysts

Catalyst	Mixed anions	Electrolyte	Substrate	Overpotential (mV) HER (10 mA cm^−2^)	Overpotential (mV) OER (10 mA cm^−2^)	Tafel slope (mVdec^−1^)	Durability	Refs.
CoO_*x*_S_0.18_	OS	0.1 M KOH	Glassy carbon	375	–	200	–	[50]
A-CoS_4.6_O_0.6_ PNCs	OS	1 M KOH 0.1 M PBS	Glassy carbon	–	290 270 (onset)	67 164	–	[48]
NiFeS-2	OS	0.1 M KOH	Glassy carbon	281	286	56	3 h	[59]
FNHNs/NF	OS	1 M KOH	Ni foam	140	340	82 & 69	10 h	[138]
NiCo_2_O_*x*_S_4−*x*_	OS	1 M KOH	FTO	–	370	43	24 h	[52]
NiCoS_0.14_O_3.25_ NSs/NF	OS	1 M KOH	Ni foam	170	250	44	6 h	[136]
S–CoO_*x*_	OS	1 M KOH	Ni foam	136	370	80 & 109	50 h	[128]
Cu@Cu_2_S@NiCoO_2−*x*_S_*x*_ NWs	OS	1 M KOH	Copper foam	203 (20 mV cm^−2^)	295 (50 mA cm^−2^)	63 & 50	30 h & 24 h	[137]
MoP|S	PS	0.5 M H_2_SO_4_	Ti foil	64	–	–	1000 cycles	[148]
rGO-PdPS	PS	0.5 M H_2_SO_4_	Glassy carbon	90	–	46	1000 cycles	[149]
rGO-supported few-layer FePS_3_	PS	0.5 M H_2_SO_4_	Glassy carbon	50	–	54	–	[150]
Co NPlsPS	PS	0.5 M H_2_SO_4_	Glassy carbon	48	–	56	36 h	[68]
P-doped Ni–Fe–S microspheres	PS	1 M KOH	Glassy carbon	–	264	48	50 h	[151]
Co S|P/CNT	PS	0.5 M H_2_SO_4_	Carbon fiber	48	–	55	100 h	[216]
Co_0.9_S_0.58_P_0.42_	PS	0.5 M H_2_SO_4_ 1 M KOH	Glassy carbon	139	266	69 & 48	20 h	[64]
NiCoPS/CC	PS	1 M KOH 0.5 M H_2_SO_4_	Carbon cloth	57	230	45 & 62	40 h	[141]
NiP_0.62_S_0.38_	PS	1 M KOH	Ni foam	52	240	52.3 & –	200 h	[65]
Ni_0.95_Co_0.05_PS_3_	PS	1 M KOH	Glassy carbon	77	–	71	–	[217]
FeP|S/CNT FeS|P/CNT	PS	0.5 M H_2_SO_4_	Carbon fiber	88 130	–	36 45	–	[218]
NiFeSP–NF	PS	1 M KOH	Ni foam	94	240@50 mA cm^−2^	83 & 76	25 h	[219]
WS_2(1−*x*)_P_2*x*_ NRs	PS	0.5 M H_2_SO_4_	Carbon fiber	98	–	71	25 h	[152]
Ni_0.9_Fe_0.1_PS_3_	PS	1 M KOH	Glassy carbon	72	329@20 mA cm^−2^	73 & 69	50 h	[66]
CoPS/CP	PS	0.5 M H_2_SO_4_	Carbon paper	26	–	43	100 h	[143]
NiCoP|S /CC	PS	0.5 M H_2_SO_4_ 1 M KOH	Carbon cloth	90 76	–	52 65	1000 cycles	[220]
NiFePS_3_	PS	1 M KOH	Glassy carbon	–	223	42	2000 cycles	[221]
CoPS/N–C	PS	0.5 M H_2_SO_4_ 1 M KOH	Glassy carbon	80 148	–	68 78	16 h	[144]
NiPS_3_ NSs-G-1:1& 2 mg NiPS3	PS	1 M KOH	Glassy carbon	–	294	43	30 h	[153]
CoP@PS/NCNT	PS	0.5 M H_2_SO_4_	NCNT	80	–	53	4 h	[222]
FeCoNiPS	PS	1 M KOH	Carbon paper	284	162	41&43	18 h	[154]

**Table 3:** Comparison of the OER and HER performance of typical mixed chalcogenides (sulfoselenides, selenotellurides, hydroxy (sulfates, sulfides), and phosphoselenides), (oxy)hydroxides, and borides/borates electrocatalysts

Catalyst	Mixed anions	Electrolyte	Substrate	Overpotential (mV) HER (10 mA cm^−2^)	Overpotential (mV) OER (10 mA cm^−2^)	Tafel slope (mV dec^−1^)	Durability	Refs.
WS_2(1−*x*)_Se_2*x*_	SSe	0.5 M H_2_SO_4_	Glassy carbon	80	–	85	67 h	[161]
WS_2(1–*x*)_Se_2*x*_/NiSe_2_	SSe	0.5 M H_2_SO_4_	Ni foam	88	–	46.7	1000 cycles	[165]
MoS_2(1_–_*x*)_Se_2*x*_	SSe	0.5 M H_2_SO_4_	Glassy carbon	80–100	–	45–55	10,000 cycles	[157]
WS_2(1−*x*)_Se_2*x*_(S NRs–Li)	SSe	0.5 M H_2_SO_4_	Carbon fibers	173	–	68	6 h	[223]
Co(S_0.73_Se_0.27_)_2_	SSe	0.5 M H_2_SO_4_	Carbon fiber paper	157	–	45	20 h	[224]
MoS_2(1−*x*)_Se_2*x*_ (X-0.61)	SSe	0.5 M H_2_SO_4_	Glassy carbon	279	–	106	22 h	[225]
W(Se_0.4_S_0.6_)_2_-C-10	SSe	0.5 M H_2_SO_4_	Carbon fiber mats	174	–	106	12 h	[226]
Mo(S_0.53_Se_0.47_)_2_	SSe	0.5 M H_2_SO_4_	Carbon cloth	183	–	56	2000 cycles	[227]
MoSSe@rGO	SSe	0.5 M H_2_SO_4_	Glassy carbon	135 @ 5 mA cm^−2^	–	51 @ 5 mA cm^−2^	5000 cycles	[26]
Mo_0.3_W_0.67_S_1.33_Se_0.67_	SSe	0.5 M H_2_SO_4_	Glassy carbon	93	–	55	5000 cycles	[167]
CoS_2_Se_2(1−*x*)_	SSe	0.5 M H_2_SO_4_	Carbon fiber	130	–	44	1000 cycles	[164]
MoS_2_Se_2(1−*x*)_ (X-0.54)	SSe	0.5 M H_2_SO_4_	Glassy carbon	219	–	55	1000 cycles	[72]
MoS_2(1−*x*)_Se_2*x*_/NiSe_2_	SSe	0.5 M H_2_SO_4_	NiSe_2_ foam	69	–	42	16 h	[228]
WS_2(1−*x*)_Se_2*x*_/ NiSe_2_	SSe	0.5 M H_2_SO_4_	NiSe_2_ foam	88	–	47	8 h	[228]
Co(S_0.71_Se_0.29_)_2_ Co(S_0.22_Se_0.78_)_2_	SSe	1 M KOH	Ni Foam	122	283	86 & 66	20 h	[229]
MoS_2(1−)_Se_2*x*_ WS_2(1−*x*)_Se_2*x*_	S,Se	0.5 M H_2_SO_4_	FTO	141 167	–	67 107	20 h	[166]
WSe_*x*_Se_1−*x*_–15 NPA	SSe	0.5 M H_2_SO_4_	W foil	110	–	59	6 h	[230]
Fe_4.5_Ni_4.5_S_8−*y*_Se_*y*_	SSe	0.5 M H_2_SO_4_	Brass rod	172	–	40	–	[168]
P/Se–MoS_2_/CFP	SSeP	0.5 M H_2_SO_4_	Carbon fiber paper	93	–	50	10 h	[158]
MoSe_0.12_Te_1.79_	SeTe	0.5 M H_2_SO_4_	Graphite	410	–	62	–	[170]
ZCS-30	OHSO_4_	0.5 M KOH	Glassy carbon	–	370	60	11 h	[171]
NiCo_2_(SOH)_*x*_	OHS	1.0 M NaOH	Ni foam	–	290	47	30 h	[61]
NiFeLDH OHS	OHS	0.1 M KOH	Glassy carbon	–	286	82	6 h	[172]
P–NiSe_2_@N-CNTs/NC	PSe	1 M KOH	Glassy carbon	95	306	82 & 61	2000 cycles	[73]
EG/H–Co_0.85_-Se|P	PSe	1 M KOH	EG foil	150	–	83	10 h	[176]
CoP_1.37_Se_0.63_NWs	PSe	0.5 M H_2_SO_4_ 1 M KOH	Carbon fiber	70 98	–	54	12 h	[175]
CoOSeP@Co foil	PSe	1 M KOH	Co foil	155	347	76 & 74	30 h	[231]
FeCoCr/GC	OOH	0.1 M KOH	Glassy carbon	–	490	60	40,000 s	[199]
CoOOH NS	OOH	1 M KOH	Glassy carbon	–	300	38	12 h	[232]
FeCoW	OOH	0.1 M KOH	Gold foam	–	191	–	500 h	[7]
Fe–OOH/NF-200	OOH	1 M KOH	Ni foam	–	290	48	11 h	[51]
Fe–CoOOH/G	OOH	1 M KOH	Glassy carbon	–	330	37	5.5 h	[233]
W_0.5_Co_0.4_Fe_0.1_/NF	OOH	1 M KOH	Ni foam	–	310@ 100 mA cm^−2^	32	510 h	[234]
NiFeOOH/EG	OOH	1 M KOH	Exfoliated graphite	–	214	21	100 h	[235]
Ni(OH)/ FeOOH	OOH	1 M NaOH	FTO	–	300	–	50 h	[193]
Ir-Doped Ni-(Oxy)hydroxide	OOH	1 M KOH	Glassy carbon	–	270	45.2	20 h	[196]
Fe–CoNi–OH	OOH	1 M KOH	Ni foam	–	210	28.0	108 h	[198]
CoCuFeMoOOH@Cu	OOH	1 M KOH	Cu foil	–	199	48.8	72 h	[201]
CoV–UAH	OOH	1 M KOH	Au foam	–	215	44	170 h	[202]
Ni_70_Fe_30_(H)	OOH	0.1 M KOH	Glassy carbon	–	292	30	2 h	[236]
Elox H_2_S NF	OOH	1 M KOH	Ni foam	–	256	41	10 h	[237]
Ni–Fe–SS–NC	OOH	1 M KOH	Stainless steel	–	232	51	340 h	[194]
FeNiO_*x*_H_*y*_	OOH	1 M KOH	Ni foam	–	206	39	50 h	[195]
Fe_0.03_Co_0.67_OOHPNSAs/CFC	OOH	1 M KOH	Carbon fiber cloth	–	266	30	24 h	[197]
Ni_3_Ge_2_O_5_(OH)_4_	OOH	1 M KOH	Glassy carbon	–	320	68	4.5 h	[238]
1 T–MoS_2_/Ni^2+^OOH_2−_	OOH	1 M KOH	Carbon fiber paper	73	–	75	30 h	[239]
Zn_0.2_Co_0.8_OOH	OOH	1 M KOH	Glassy carbon	–	235	34.7	40 h	[49]
Co_1.8_Ni(OH)_5.6_@Co_1.8_NiS_0.4_(OH)_4.8_	OH–OHS	0.1 M KOH	Glassy carbon	–	274	45	2.5 h	[240]
Ni_*x*_Fe_1−*x*_–AHNAs	OOH-alloy	1 M KOH	Ni foam	–	190	21	120 h	[241]
Ni–Bi–Pi/CC	BP	0.1 M KBi	Carbon cloth	–	440	139	23 h	[208]
Fe–Pi–Bi/CC	BP	0.1 M KBi	Carbon cloth		434	89	20 h	[209]
Co–B–P/NF	BP	1 M KOH	Ni foam	42	–	42.1	20 h	[74]
Co–P–B/CP	BP	0.5 M H_2_SO_4_	Carbon paper	–	–	62–68	–	[207]
NiVFe–B–P LDHs@NF	BP	1.0 M KOH	Ni foam	117	–	68	24 h	[210]

**Fig. 16:**
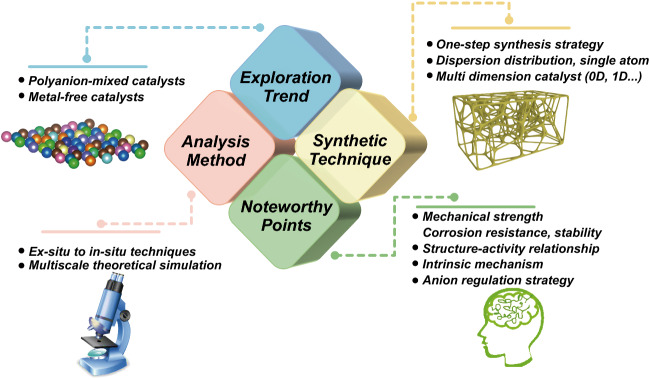
Outlook and perspective research directions for anion-mixed catalysts served for water electrolysis

Although numerous reports have been investigated in using anion-mixed electrocatalysts for water hydrolyzer, it still has much more possibility for further improvement. Many studies only focus on improving performance and neglect the exploration of the mechanism, which has no reference significance for other researchers. Besides, the synthesis method is too single, and the research model is gradually becoming routine and single, without considering the requirements of actual production and application. Furthermore, innovative and challenging works are rare. Given these, future research directions (Fig. 16) can be presented as follows:

(i) Polyanion-mixed and metal-free catalyst: Recently, polyanion-mixed catalysts have gradually become a research hotspot, and tri-anion-mixed such as SPO have been displayed [117]. Different anions have different electronegativity, and their effective combination can meet the diversified requirements of material properties. The synergy will significantly stimulate the intrinsic activity of the catalyst. Then, the development of anion-mixed with absolute metal-free nanocomposite (i.e., g-C_3_N_4_/graphene, N-doped carbon, C_3_N_4_ quantum dots on graphene) [242–244] is a further possible approach to improving electrical conductivity, reducing costs, and promoting durability of water-splitting activity.
(ii) Progressive strategies: Single-atom catalysts (SACs) have demonstrated superior performance for electrocatalysts [245]. Drawing on SACs advantages, dispersing mixed anions and avoiding agglomeration as much as possible will be one of the promising ways to tune the water-splitting performance. Besides, for dimensional regulation, the Carbon quantum dots (CQDs) has possessed many unique advantages such as high electron‐transfer abilities, abundant, non-toxic, and low cost [246]. Moreover, the surface of CQDs has many abundant functional groups (–OH, –COOH, –NH_2_, etc.), which offer sufficient active sites for constructing electroactive catalysts. From these significant advantages, combining mixed anions with CQDs is an efficient method to fabricate advanced electrocatalysts for water-splitting. Furthermore, one-step synthesis strategies are urgently needed to improve production efficiency.
(iii) Advanced characterizations: Catalytic reaction is a dynamic process, and advanced characterization techniques (i.e., in-situ XRD, Raman, TEM) are potent means to clarify the reaction pathway and internal mechanism. The clarification of these issues can in turn guide material design, thereby shortening the development cycle. Besides, computer simulations (DFT calculations, machine learning, molecular dynamics simulations (MD), etc.) can effectively assist in verifying conjectures about the mechanism and expanding the library of advanced materials, thereby greatly reducing the cost of trial and error and correcting the traditional cooking research mode.
(iv) Focus on core issues: Except for meeting laboratory conditions, catalysts that are close to actual production needs are more worthy of researchers' attention. Anion-mixed catalysts are mostly low-dimensional nanostructures, and it is necessary to construct a series of reasonable evaluation indexes such as service life, mechanical strength, and corrosion resistance. Besides, many catalysts have attractive appearance structures. The exploration of their formation mechanism and even the atomic level structure–activity relationship is of great importance. Moreover, the internal mechanism that anion-mixed-promoted catalytic activity urgently needs more intuitive physical models to explain, rather than perceptual understanding. Theoretical explorations need to be strengthened, such as LOM mechanism and the diversified influence of anion mixing on electron transfer. Finally, anion regulation strategy deserves more attention. Accurate regulation at the atomic scale will be more required to achieve the unique coordination environment and intrinsic catalytic improvement.
